# Revealing the Mechanisms of Shikonin Against Diabetic Wounds: A Combined Network Pharmacology and In Vitro Investigation

**DOI:** 10.1155/jdr/4656485

**Published:** 2025-03-10

**Authors:** Meng Tian, Junchao Wu, Qian Du, Jiale Han, Meng Yang, Xiang Li, Mingzhu Li, Xiaofeng Ding, Yeqiang Song

**Affiliations:** ^1^First College of Clinical Medical, Shandong University of Traditional Chinese Medicine, Jinan, Shandong, China; ^2^Shanghai Skin Disease Hospital, Tongji University School of Medicine, Shanghai, China; ^3^Stomatology Hospital Affiliated to Tongji University, Tongji University, Shanghai, China; ^4^Department of Cosmetic Dermatology, The Affiliated Hospital of Shandong University of Traditional Chinese Medicine, Jinan, Shandong, China; ^5^Department of Emergency Medicine, Shanghai Jiao Tong University Affiliated Sixth People's Hospital, Shanghai, China

## Abstract

**Background:** Shikonin (SHK) possesses extensive pharmacological effects including antimicrobial and anti-inflammatory properties for diabetic wound (DW), while its molecular mechanism remains to be clarified. In this study, we investigated the potential mechanisms of SHK in treating DW by combining network pharmacology and in vitro experiments.

**Methods:** We obtained potential targets for SHK and DW from the publicly available database. Based on the interaction network and conducting GO and KEGG pathway enrichment analysis, we constructed a target pathway network to explore the relationship between SHK and DW. To validate the mechanism of SHK, we established an in vitro experimental model.

**Results:** Sixty intersecting targets between SHK and DW were obtained, and the top 10 targets of the protein–protein interaction (PPI) network included AKT1, SRC, EGFR, CASP3, MMP9, PPARG, ESR1, ANXA5, MMP2, and JAK2. Based on target–pathway networks, the PI3K-AKT signaling pathway was found to be a signaling pathway with low *p* value in enrichment analysis. In vitro experiments revealed that SHK significantly promoted angiogenesis. Meanwhile, SHK could inhibit the high glucose-induced human umbilical vein endothelial cell dysfunction through regulating the PI3K-AKT pathway.

**Conclusion:** This study initially revealed the molecular mechanism of SHK in DW by multitarget and multipathway. The PI3K–AKT signaling pathway, MAPK signaling pathway, and AGE-RAGE signaling pathways may be the main pathways of SHK in treating DW.

## 1. Introduction

Diabetic wound (DW), as one of the most severe complications of diabetes, causes substantial health and economic burdens globally. Even after healing, the 1-year recurrence rate for DW still reaches 40%, making their treatment a notably challenging endeavor [1, 2]. In contrast to acute wounds, which undergo the stages of coagulation, inflammation, proliferation, and remodeling, DWs are marked by hyperglycemia, elevated inflammation, and sustained hypoxemia [3, 4]. These characteristics disrupt the customary healing trajectory, leading to a halt at various phases. Moreover, hyperglycemia can induce vascular constriction, alterations in erythrocyte membranes, and a deficient oxygen supply to wound tissues [5]. The complex pathological factors associated with DW significantly complicate the healing process. In clinical treatment, reducing blood sugar is a component of DW patient treatment, but these measures are insufficient for comprehensive DW management [6]. Although novel hydrogel wound dressings have emerged in recent years, hydrogels remain limited as effective therapeutic options. This limitation arises from the inability of single-dimensional hydrogels to address the complex and dynamic microenvironment of DW, while composite hydrogels often present biosafety concerns [7].

Currently, phytotherapy is widely used worldwide as an approach to identify new active compounds from natural sources for treating heterogeneous and chronic diseases [8]. Medicinal plants, with their lower toxicity and higher patient compliance, represent a promising source of candidate drugs for DW [9]. However, current treatments for DW have limitations, particularly in reducing infection and alleviating chronic inflammation [10]. Therefore, it is important to explore new natural sources for DW therapy. Shikonin (SHK), the main bioactive ingredient of *Lithospermum erythrorhizon* (Zicao) in traditional Chinese medicine, possesses a variety of pharmacological properties, including antimicrobial, anti-inflammatory, antioxidant effects, promotion of cell proliferation, and acceleration of wound healing [11]. These pharmacological characteristics make SHK a promising therapeutic agent for treating chronic wounds such as DW. The role of SHK in wound healing is primarily reflected in the following aspects.

First, SHK exhibits potent antimicrobial activity, inhibiting the growth of pathogenic microorganisms, such as *Staphylococcus aureus*, thereby reducing the risk of infection at the wound site and creating a favorable environment for healing [12]. Second, SHK plays a critical role in accelerating wound healing by promoting the migration and proliferation of skin cells through the induction of epithelial–mesenchymal transition (EMT) [13]. Localized hyperglycemia suppresses normal cellular functions, while SHK can enhance the expression of EMT-related molecules, improving this impaired functionality and accelerating healing [14]. Additionally, SHK alleviates chronic inflammation at the wound site by inhibiting the nuclear factor-*κ*B (NF-*κ*B) pathway [15]. SHK also modulates the transforming growth factor-*β*1 (TGF-*β*1) pathway, reducing excessive collagen production, thus minimizing scar formation and improving the skin quality post healing [16, 17]. Through the ERK–Smad pathway, SHK downregulates the expression of *α*-smooth muscle actin (*α*-SMA), inhibiting excessive collagen synthesis and effectively reducing the abnormal scarring commonly observed in DW [18]. These findings provide evidence for the efficacy of SHK as a potential therapeutic approach for DW.

Network pharmacology is increasingly being adopted, facilitating a comprehensive understanding of the direct relationships between drugs or compounds, diseases, and their targets, informed by data across various databases [19]. In this study, we employed network pharmacology analysis to investigate the potential targets of SHK in the treatment of DW and identified the signaling pathways involved. Finally, we established an in vitro experimental model for validation purposes.

## 2. Materials and Methods

### 2.1. Molecular Characterization Data and Targets of Action of SHK

The 2D structure of SHK was obtained by searching for “shikonin” in the PubChem database (https://pubchem.ncbi.nlm.nih.gov/). The target of action of SHK was obtained from the PharmMapper database (https://www.lilab-ecust.cn/pharmmapper/) and the Comparative Toxicogenomics Database (https://ctdbase.org/).

### 2.2. Obtaining Disease-Related Targets

Disease-related targets were obtained from five databases by searching “Diabetic wounds” and “Diabetic foot ulcers (DFU)”. These included the DisGeNET database (http://www .http://disgenet.org/home/), the OMIM database (http://www.omim.org), the GeneCards database (https://www.genecards.org/), the DrugBank database (https://go.drugbank.com/), and the TTD database (http://db.idrblab.net/ttd/). After combining the data from the five databases and removing duplicate values, the data were compared in the UniProt database (https://www.uniprot.org/) to confirm the standardized names of all targets. The data for the relevant targets of SHK and DW were obtained from free, publicly available databases, and unrestricted use was permitted.

### 2.3. Construction of Protein–Protein Interaction (PPI) Network and Identification of Key Targets

After obtaining the relevant targets of SHK and the disease, a Venn diagram was utilized to show that the overlapping parts were the intersecting genes of the two, which were considered as the potential targets of action when SHK treats DW. To further demonstrate the interaction relationship between the intersecting genes, all intersecting genes were imported in the STRING database (https://cn.string-db.org/) with limited species = *Homo sapiens*, minimum required interaction score = 0.4, to generate the PPI network. The results were imported into Cytoscape (Version 3.9.1) software. The maximum neighborhood component (MNC) scores of all targets were calculated using the CytoHubba plug-in, and the targets with the top 10 scores were taken as hub genes of the PPI network.

### 2.4. Biological Pathway Enrichment Analysis

Kyoto Encyclopedia of Genes and Genomes (KEGG) and Gene Ontology (GO) enrichment analyses were used to explore possible biological mechanisms when SHK acts on DW. KEGG pathway enrichment analysis was performed using the Metascape database (https://metascape.org/) with the following screening criteria: species = *Homo sapiens*, min overlap = 3, min enrichment = 1.5, and *p* < 0.01. GO enrichment analysis including biological process (BP), cellular composition (CC), and molecular function (MF) was performed using the “clusterProfiler” program package of R software. All results were visualized using the “ggplot2” package. KEGG pathway enrichment analysis showed 25 pathways associated with the disease, and GO enrichment analysis showed the top 10 terms with *p* values in each section.

### 2.5. Explore Pathway–Target Networks

In order to demonstrate the connection between biological pathways closely related to DW, a “pathway–target” network was constructed. Cytoscape software was used to display all the targets of the five pathways with the smallest *p* value in the KEGG pathway enrichment analysis, and to observe the target interactions between different pathways. The KEGG pathway, which is closely related to DW, was displayed separately, and the involvement of hub genes in this pathway was also observed.

### 2.6. Molecular Docking

In the PubChem database (https://pubchem.ncbi.nlm.nih.gov/), a selected 3D structure of alkannin, the active ingredient (SHK), was downloaded. The structure file was converted to mol2 format using the OpenBabel 2.4.1 software. Subsequently, the small ligand molecules were optimized using the AutodockTools 1.5.6 software, and the optimized structures were obtained after hydrogenation of atoms and charge loading. These structures were saved as pdbqt files for molecular docking. The stable conformation with the lowest binding energy was selected for visualization and imported into the PyMOL 2.4 software for 3D molecular structure map construction.

### 2.7. Cell Culture

Human umbilical vein endothelial cell (HUVECs) were purchased from the National Collection of Authenticated Cell Cultures (Shanghai, China). The normal culture condition for HUVECs is low-glucose (5.5 mM) medium with 10% fetal bovine serum (FBS) (Gibco, USA). The high-glucose (HG) culture condition for HUVECs is HG (40 mM) medium with 10% FBS (Gibco, USA). The 40-mM D-glucose was chosen to mimic diabetic conditions in vitro.

### 2.8. Cell Counting Kit-8 (CCK-8) Assay

CCK-8 assay kit was used to detect the effects of different concentrations of SHK on cell viability. Different concentrations (0, 0.25, 0.5, 1, 2, and 4 *μ*M) of SHK were added to 96-well plates for 24 h. Take 10 *μ*L of the CCK-8 solution and add it to each well. Incubate for 1 h and then measure the optical density at 450 nm. Based on the detection results, the optimal concentration of SHK was obtained for subsequent experiments.

### 2.9. Scratch Test

Six well plates were used for the scratch test. The key to the cell inoculation technique is to have as uniform a number as possible and to achieve 100% fusion after 24 h. A scratch was then made on the cell layer using a sterile 200-*μ*L pipette tip. Next, the width of the scratch was rinsed with phosphate-buffered saline (PBS) and photographed using a microscope. The cells were incubated at 37°C for 24 h before photographing the width of the scratch using a microscope, and the images were analyzed using ImageJ software.

### 2.10. Immunofluorescence Analysis

HUVECs were cultured in 24-well plates according to different groups of interventions for 24 h. The next day, they were fixed using 4% paraformaldehyde and subsequently washed with PBS before being closed with donkey serum for 30 min. The primary antibody (VEGF; 1:200; Cell Signaling Technology) was coincubated with HUVECs overnight at 4°C. After washing with PBS, the secondary antibodies were added and incubated for 60 min at 37°C protected from light. HUVECs were observed using a fluorescence microscope (Olympus, Tokyo, Japan).

### 2.11. Quantitative Real-Time Polymerase Chain Reaction (qRT-PCR)

HUVECs from different treatments were preserved in RNA tissue stabilization solution (Beyotime, Shanghai, China). Total RNA was extracted using an RNA extraction reagent (ServiceBio, Wuhan, China) and reverse transcribed into cDNA using a PrimeScript RT Real-time Master Mix (TaKaRa, Tokyo, Japan). TB Green amplification primers (Takara Bio, Shiga, Japan) were employed for the amplification process. Real-time fluorescent quantitative PCR was then conducted using the LightCycler480 system (Roche) as the platform of choice. The comparative Ct (2^−ΔΔ*Ct*^) method was employed for data analysis, with GAPDH used as the internal control for normalization. The primer sequences can be found in Table S1.

### 2.12. Western Blotting

Radioimmunoprecipitation assay (RIPA) buffer (Thermo Scientific, USA) was used to lyse different groups of HUVECs, and then quantify the protein using the bicinchoninic acid (BCA) assay kit (Beyotime). Separate proteins using sodium dodecyl sulfate-polyacrylamide gel electrophoresis (SDS-PAGE) and transfer them into polyvinylidene fluoride membrane (Millipore, USA). Seal with 5% skim milk, then wash with Tris-buffered saline with Tween-20 (TBST) and add the following primary antibodies: CD31 (A2104; 1:1000; ABclonal), VEGF (ab1316; 1:2000; Abcam), *β*-actin (AC038; 1:10000; ABclonal), p-PI3K (ab302958; 1:2000; Abcam), PI3K (ab140307; 1:2000; Abcam), p-AKT (ab38449; 1:1000; Abcam), AKT (ab8933; 1:1000; Abcam), p-mTOR (ab109268; 1:1000; Abcam), and mTOR (ab137133; 1:1000; Abcam). The primary antibody was incubated with polyvinylidene fluoride membrane overnight at 4°C; the membrane was washed with TBST and incubated with horseradish peroxidase-conjugated secondary antibody for 1 h. Finally, the bands were detected using an enhanced chemiluminescence (ECL) kit (P1010, Applygen, Beijing, China) and a multifunctional imaging system (Tanon 5200, Shanghai, China) to detect the bands, and then the images were analyzed with ImageJ software.

### 2.13. Statistical Analysis

Data in this study are presented as mean ± SEM and analyzed by one-way ANOVA. Subsequently, multiple comparisons were performed with Tukey's post hoc test using SPSS 22.0 software. A *p* value of less than 0.05 was considered to indicate statistical significance.

## 3. Results

### 3.1. Potential Targets of SHK and DW

**Figure 1:**
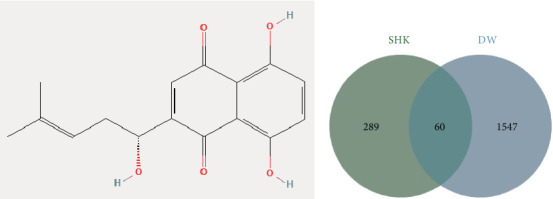
2D structure of SHK with intersecting genes for SHK treatment of DW. (a) 2D structure of SHK. (b) Venn diagram of intersecting genes of SHK-related targets and DW-related targets. SHK: shikonin; DW: diabetic wound.

The 2D structure (Figure 1(a)) of SHK were obtained from the PubChem database. Two hundred and forty-nine relevant targets of SHK were obtained from the PharmMapper database and 100 relevant targets of SHK were obtained from the Comparative Toxicogenomics Database (Tables S2 and S3). The targets for DW were obtained from and merged from five databases, where the results from the GeneCards database were taken from the top 25% of genes based on relevance scores. The results from the five databases were merged to remove duplicate values, and a total of 1611 targets were obtained (Table S4). The presentation of the two using Venn diagrams revealed 60 intersecting genes (Figure 1(b)) [20].

### 3.2. GO and KEGG Enrichment Analysis

**Figure 2:**
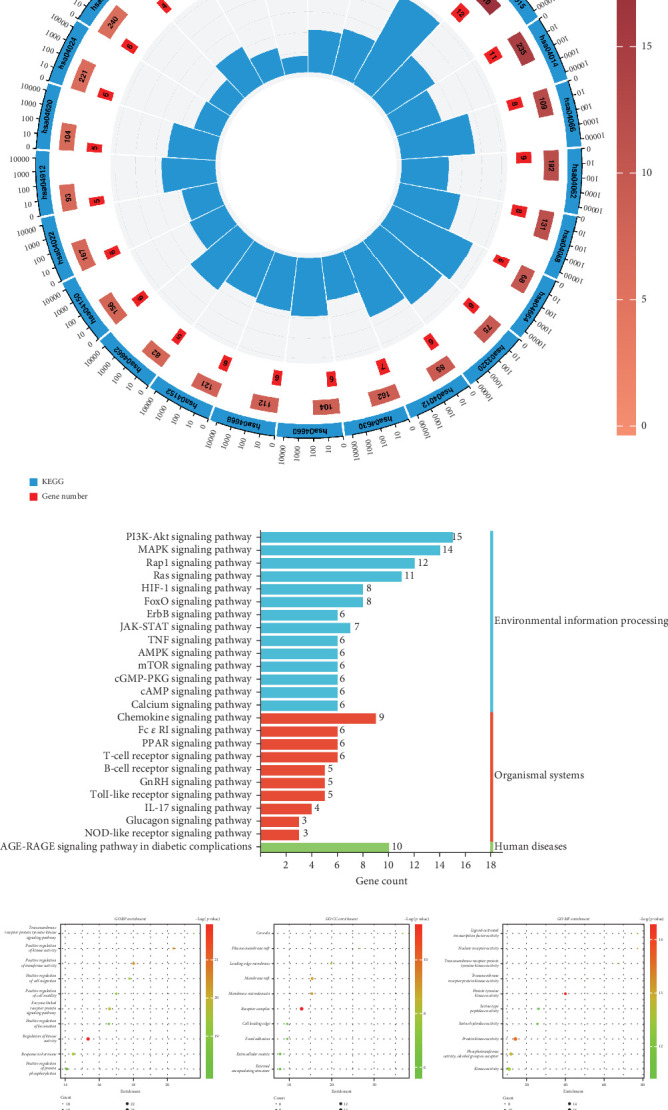
Enrichment analysis. (a) KEGG enrichment analysis circle diagram. From outside to inside: The outermost circle is the total number of genes of all pathways; the second circle is the signaling pathway number; the third circle is the number of genes contained in the pathway, and the color corresponds to the *p* value; the fourth circle is the number of intersecting genes annotated to the pathway; and the fifth circle is the ratio of the number of genes annotated to the pathway to the total number of concatenated genes of all pathways. (b) Bar graph of KEGG enrichment analysis. The horizontal coordinate is the number of genes annotated to the pathway, the vertical coordinate is the name of the pathway, and the right side is the corresponding KEGG classification of the pathway. (c) Bubble plot of GO enrichment analysis. The GO analysis includes BP, CC, and MF. The horizontal coordinate is the enrichment score; the vertical coordinate is the name of the term; the color of the dots corresponds to the *p* value; and the size of the dots corresponds to the number of genes annotated to this belonging. KEGG: Kyoto Encyclopedia of Genes and Genome; GO: Gene Ontology; BP: biological process; CC: cellular composition; MF: molecular function.

KEGG pathway enrichment analysis was performed using Metascape database. Sixty intersected genes were uploaded into the database, and the species was selected as “Homo sapiens”. A total of 131 pathways were identified, and Figures 2(a) and 2(b) show 25 signaling pathways, including PI3K–AKT signaling pathway, MAPK signaling pathway, AGE-RAGE signaling pathway in diabetic complications, Rap1 signaling pathway, and RAP1 signaling pathway, Ras signaling pathway, and HIF-1 signaling pathway. GO enrichment analysis included BP, CC, and MF; the top 10 terms in terms of *p* value for each section are shown in Figure 2(c). BP enriched 919 terms, including positive regulation of protein phosphorylation, response to hormone, regulation of kinase activity, positive regulation of locomotion, and enzyme-linked receptor protein signaling pathway. CC enriched 44 terms, including external encapsulating structure, extracellular matrix, focal adhesion, cell leading edge, and receptor complex. MF enriched 83 terms, including kinase activity, phosphotransferase activity, alcohol group as acceptor, protein kinase activity, serine hydrolase activity, and serine-type peptidase activity. Detailed information on KEGG and GO enrichment analysis is shown in Table S5.

### 3.3. Establishment of PPI Networks and Identification of Hub Genes

**Figure 3:**
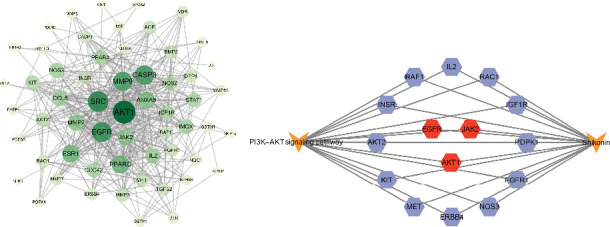
PPI network vs. hub-pathway network diagram. (a) PPI network diagram. Each point in the graph corresponds to an intersecting gene. The larger the dot, the darker the color, and the larger the font, the higher the MNC score. (b) Network diagram of the “hub gene–PI3K–AKT signaling pathway”. Hexagons are intersecting genes annotated to the pathway, and red dots are hub genes. PPI: protein–protein interaction.

After importing the 60 intersecting genes into the STRING database, all PPI networks were generated (Figure 3(a)). The results were imported into Cytoscape software, which had a total of 58 points and 402 edges in the network. After calculating the MNC scores of all targets using the CytoHubba plugin, the top 10 targets ranked according to their scores were AKT1, SRC, EGFR, CASP3, MMP9, PPARG, ESR1, ANXA5, MMP2, and JAK2. These genes are believed to be hub genes in which SHK plays a key role in the treatment of DW.

Among these hub genes, AKT1 and EGFR were particularly important. AKT1 is a central regulator in the PI3K–AKT pathway, which governs vital cellular processes such as proliferation, migration, and survival [21]. The PI3K–AKT pathway is critical for promoting cell migration, and its dysfunction can impair EMT, cell proliferation, and overall wound healing [22]. Therefore, AKT1 represents a central factor in SHK potential to enhance DW healing by regulating cell migration and survival. EGFR, as an epidermal growth factor receptor, is highly expressed in cells of wounded skin and promotes wound re-epithelialization by enhancing epithelial cell migration and proliferation [23]. Activation of EGFR initiates signaling through the PI3K–AKT–mTOR pathway, thereby supporting cell survival and neovascularization, which are crucial for improving DW healing [24]. These findings explained the importance of AKT1 and EGFR in SHK therapy in DW. The PI3K–AKT signaling pathway was found to be a signaling pathway with low *p* value in enrichment analysis, and the association of hub genes with this pathway was further demonstrated in Figure 3(b), reinforcing the significance of this pathway as SHK potential therapeutic target.

In addition, other hub genes, such as MMP9 and CASP3, were involved in tissue remodeling and the regulation of apoptosis. Proper regulation of these genes is essential for DW healing, as imbalances can lead to ineffective healing or scarring [25]. Overall, these results suggested that SHK might have promoted DW healing in a multidimensional manner by regulating these hub genes, particularly AKT1 and EGFR.

### 3.4. Molecular Docking

**Figure 4:**
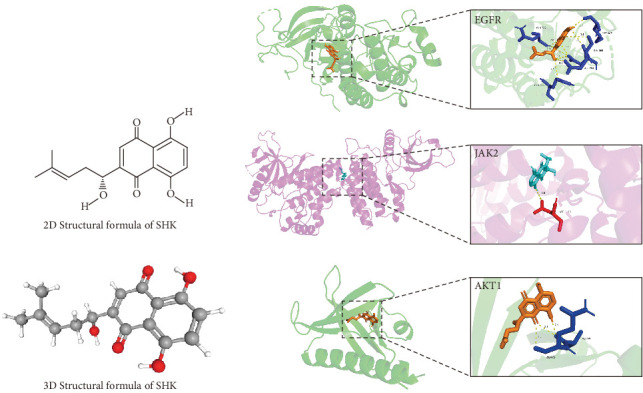
The binding mode of SHK with EGFR, JAK2, and AKT1. (a) 2D structure with SHK. (b) SHK with EGFR, JAK2, and AKT1. SHK: shikonin; EGFR: epidermal growth factor receptor; JAK2: Janus kinase 2; AKT1: serine/threonine kinase1.

Further molecular docking analysis was conducted to evaluate the binding interactions between SHK and three key target proteins (EGFR, JAK2, and AKT1). These target proteins played crucial roles in angiogenesis, cell proliferation, and migration, all of which are essential processes in DW healing. The 2D and 3D structures of SHK, as well as the core target proteins, were shown in Figure 4(a). The structures of the target proteins were EGFR (7tvd), JAK2 (2b7a), and AKT1 (1unq). The docking results of SHK with the core targets are presented in Figure 4(b), and the binding affinities are listed in Table 1. SHK is bound to EGFR (7tvd) with a binding affinity of −6.45 kcal/mol. Specifically, SHK formed multiple hydrogen bonds with five amino acid residues of EGFR (GLN-812, GLN-976, ASP-984, GLU-985, and MET-987), with 10 hydrogen bonds and relatively short bond lengths (average ~2.5 Å), suggesting that SHK could stably bind to the active site of EGFR. For JAK2 (2b7a), SHK formed a hydrogen bond with the ASP-1118 residue at a bond distance of 2.9 Å, with a binding affinity of −5.55 kcal/mol. The binding affinity of the JAK2 inhibitor ruxolitinib with JAK2 is −8.0 kcal/mol, similar to that of SHK with JAK2, which suggests that SHK activity regulates the JAK2–STAT pathway similar to JAK2 inhibitors [26, 27]. Additionally, the binding affinity of SHK with AKT1 (1unq) was −5.81 kcal/mol. SHK formed hydrogen bonds with the GLN-59 and ALA-58 residues of AKT1 at bond distances of 3.3, 2.8, and 2.4 Å, respectively. Since the AKT pathway is crucial for cell proliferation, survival, and angiogenesis in DW healing [28], these results suggested that SHK could regulate the activity of the AKT1 signaling pathway by binding to AKT1, thereby promoting wound healing.

### 3.5. SHK Promoted Angiogenesis In Vitro

**Figure 5:**
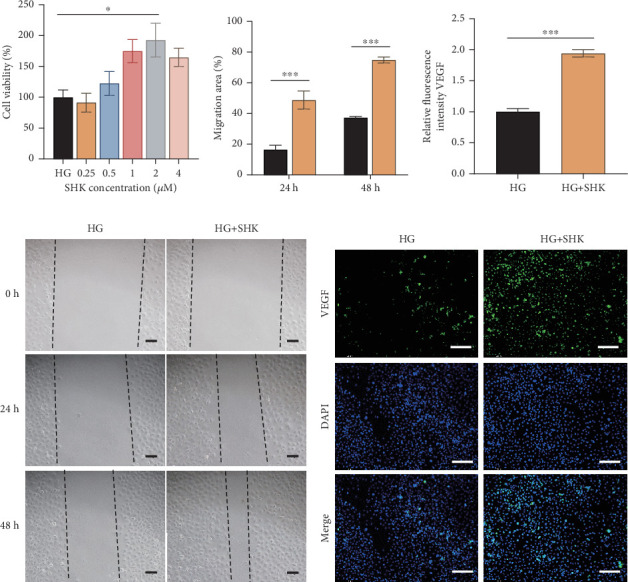
SHK promoted angiogenesis in HUVECs. (a) Effect of different concentrations of SHK solution on HUVEC activity. (b, d) Scratch assay in different groups and the change of results. (c, e) Expression of VEGF in HUVECs detected via immunofluorescence staining. Scale bar: 100 *μ*m. HG: high-glucose group, treated with high-glucose medium; HG + SHK: high-glucose + 2-*μ*M shikonin group, treated with high-glucose medium, and 2-*μ*M shikonin. ∗*p* < 0.05, ∗∗*p* < 0.01, and ∗∗∗*p* < 0.001 vs. HG.

HUVECs are differentiated endothelial cells with the ability to proliferate, migrate, and form new capillary networks [29]. In DW, endothelial dysfunction and impaired angiogenesis are key factors that delay healing [30]. HUVECs secrete various cytokines and growth factors that can accelerate angiogenesis and facilitate wound repair [31]. Therefore, they serve as a cellular model for studying DW angiogenesis and endothelial function. We investigated the mechanism of action of SHK using HUVECs. According to the CCK-8 results, the 2-*μ*M concentration of SHK had the least effect on cell activity and was therefore selected for subsequent in vitro experiments (Figure 5(a)). Under HG conditions, the excessive production of reactive oxygen species can cause damage to skin cells, such as lipid peroxidation, protein modification, and DNA damage, inhibiting cell proliferation and migration, thereby delaying wound healing [32]. As the results of the cell scratching assay, the rate of scratch recovery of HUVECs in the HG + SHK group was significantly faster than that of the HG group (Figures 5(b) and 5(d)), suggesting that SHK could promote HUVECs' migration and repair. Additionally, immunofluorescence staining further confirmed that SHK significantly enhanced the expression of VEGF (Figures 5(c) and 5(e)), indicating that SHK might accelerate wound healing by promoting angiogenesis and upregulating VEGF expression. These findings suggested that SHK could alleviate HG-induced oxidative stress and regulate the expression of angiogenesis-related growth factors such as VEGF, thereby promoting HUVECs' proliferation and migration and accelerating the process of DW.

### 3.6. SHK Inhibited the HG-Induced HUVEC Dysfunction Through Regulating the PI3K–AKT Pathway

**Figure 6:**
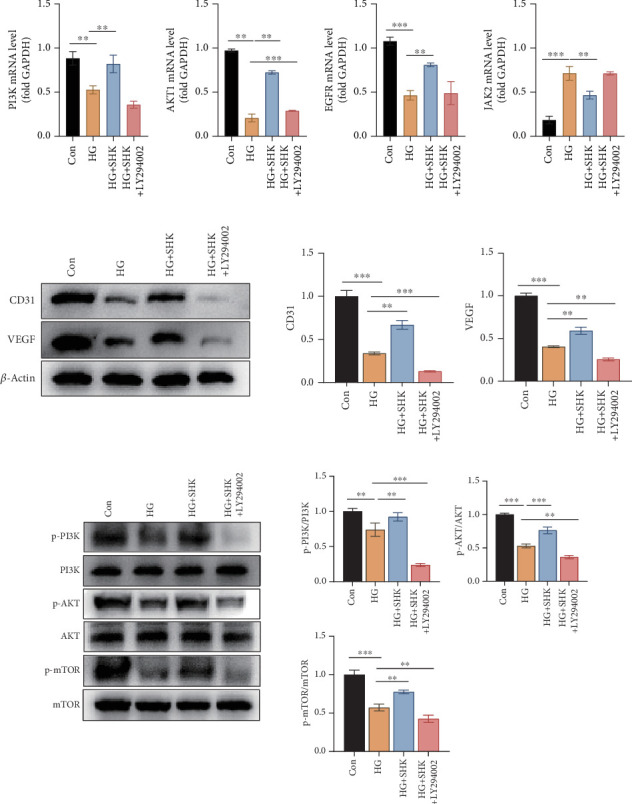
SHK promoted angiogenesis through regulating the PI3K–AKT pathway. (a) The gene expression of PI3K, AKT1, EGFR, and JAK2 in different groups. (b) The protein expression of CD31 and VEGF in different groups. (c) The protein expression of p-PI3K, PI3K, p-AKT, AKT, p-mTOR, and mTOR in different groups. Con: control group, treated with low-glucose medium; HG: high-glucose group, treated with high-glucose medium; HG + SHK: high-glucose + 2-*μ*M shikonin group, treated with high-glucose medium and 2 *μ*M shikonin; HG + SHK + LY294002: high-glucose + 2-*μ*M shikonin + LY294002 group, treated with high-glucose medium, 2 *μ*M shikonin, and 10 *μ*M LY294002. ∗*p* < 0.05, ∗∗*p* < 0.01, and ∗∗∗*p* < 0.001 vs. HG.

The activation of the PI3K–AKT pathway can accelerate DW healing by upregulating key growth factors such as VEGF, FGF, and EGF [33], enhancing endothelial cell proliferation, migration, and angiogenesis, and remodeling extracellular matrix (ECM) [34]. To further investigate the effect of SHK on the PI3K–AKT pathway, we used the PI3K inhibitor (LY294002) to intervene HUVECs. The qRT-PCR results showed that SHK could activate the PI3K–AKT pathway and enhance the expression of EGFR genes, both of which were suppressed under HG conditions. At the same time, SHK inhibited the HG-induced expression of the JAK2 gene (Figure 6(a)). Intervention with LY294002 significantly suppressed the activating effects of SHK on the PI3K–AKT pathway and EGFR, while also inhibiting SHK's suppressive effect on JAK2 activation.

Under HG conditions, the expression of angiogenesis-related proteins, CD31 and VEGF, was significantly downregulated in HUVECs. However, SHK intervention significantly promoted the expression of both CD31 and VEGF (Figure 6(b)). After LY294002 intervention, the aforementioned effects of SHK were reversed. Additionally, HG significantly downregulated the expression levels of p-PI3K and p-AKT in HUVECs, while SHK intervention upregulated the expression of both. In the HG + SHK + LY294002 group, the phosphorylation levels of the PI3K–AKT pathway–related proteins were significantly inhibited (Figure 6(c)).

HG inhibited the activation of the PI3K–AKT pathway, leading to impaired angiogenesis and delayed wound healing. SHK activated this pathway, restoring the expression of angiogenesis-related factors such as CD31 and VEGF, thereby promoting HUVECs' migration and proliferation and improving the healing process of DW. Furthermore, SHK suppressed the activation of JAK2, further modulating cell signaling involved in inflammation and wound repair. These results suggested that the PI3K–AKT pathway might be a key mechanism by which SHK promoted DW healing.

## 4. Discussion

Diabetes mellitus correlates with the damage of various organs and tissues, with prolonged hyperglycemia identified as a primary factor complicating the wound healing process [35]. Hyperglycemia triggers protein glycation and the generation of superoxide radicals, resulting in heightened oxidative stress and delayed wound healing [36]. Therefore, it is crucial to find effective drugs to promote DW healing. SHK exhibits extensive pharmacological effects and is applicable in the treatment of infections, inflammation, and cancer [37–41]. The PI3K–AKT signaling pathway is a fundamental signaling pathway that regulates cell proliferation, differentiation, development, and apoptosis; and several studies have reported the involvement of the PI3K–AKT pathway in the disease progression and treatment of DW [42–45]. In diabetes, SHK can activate the AKT pathway to inhibit apoptosis and oxidative stress induced by hyperglycemia [46]. Previous research has demonstrated that composites containing SHK can activate the PI3K–AKT pathway to ameliorate inflammatory infiltration and impaired proliferation in DW [47]. In addition, SHK-containing nanofibers have better antimicrobial, antioxidant, and biocompatibility properties and promote DW healing by upregulating growth factors (CD31 and HIF-1*α*) and downregulating inflammatory factors (CD68) [48]. In vivo experiments found that SHK can increase the activity of fibroblast nuclear endothelial cells, reduce inflammatory infiltration, increase collagen deposition, promote angiogenesis, and thus accelerate wound healing [49]. While there were reports on the study of SHK in diabetes and its associated complications, the investigation into its effects on DW required further exploration.

In this investigation, network pharmacology analysis identified that EGFR, JAK2, and AKT1 may be the key targets of SHK in regulating the PI3K–AKT pathway in the treatment of DW. The results of the enrichment analysis highlighted the PI3K–AKT pathway as significantly enriched, indicating its potential as a critical pathway. The hub genes (AKT1, SRC, EGFR, CASP3, MMP9, PPARG, ESR1, ANXA5, MMP2, and JAK2) of SHK for the treatment of DW were identified by PPI network. To preliminarily validate the affinity between SHK and the key targets, we employed molecular docking to simulate their binding interactions. The results demonstrated that SHK exhibited strong affinity with EGFR, JAK2, and AKT1. The molecular docking results were validated by qRT-PCR. Treatment of diabetic mice with a traditional Chinese medicine compound was found to activate the EGFR–PI3K–AKT pathway, enhancing the proliferation of keratinocytes in the wounds and facilitating the recovery of DW [45]. It was found in in vitro experiments that SHK could promote HUVECs' proliferation and migration and activate the PI3K–AKT signaling pathway. In a HG environment, there was a decrease in VEGF and CD31 expression in HUVECs. The application of SHK not only ameliorated this condition but also activated the PI3K–AKT signaling pathway.

In this study, we found that EGFR, JAK2, and AKT1 may be the key targets of SHK in regulating the PI3K–AKT pathway in the treatment of DW. Among them, EGFR is a protein tyrosine kinase receptor that can be expressed in a variety of skin cells. Skin cells are closely related to the process of wound repair [50]. In normal skin, EGFR promotes wound repair by inhibiting terminal differentiation, promoting cell survival, and regulating cell migration during epidermal morphogenesis and wound healing [51]. Topical EGFR applications are found to enhance skin wound recovery [52]. Furthermore, a bioinformatics analysis revealed a significant interaction between EGFR and autophagy-related genes in DFU, suggesting that EGFR may contribute to the progression of DFU by modulating autophagy in skin cells [53]. Furthermore, JAK2, as an important proinflammatory signal, prolongs the chronic inflammation phase in DW. SHK promoted wound healing by downregulating the JAK–STAT proinflammatory pathway [54]. In the treatment of DW, the activation of AKT1 promotes cell growth and repair and inhibits apoptosis and oxidative stress induced by hyperglycemia [55]. SHK is bound to AKT1, enhancing endothelial cell proliferation and migration.

**Figure 7:**
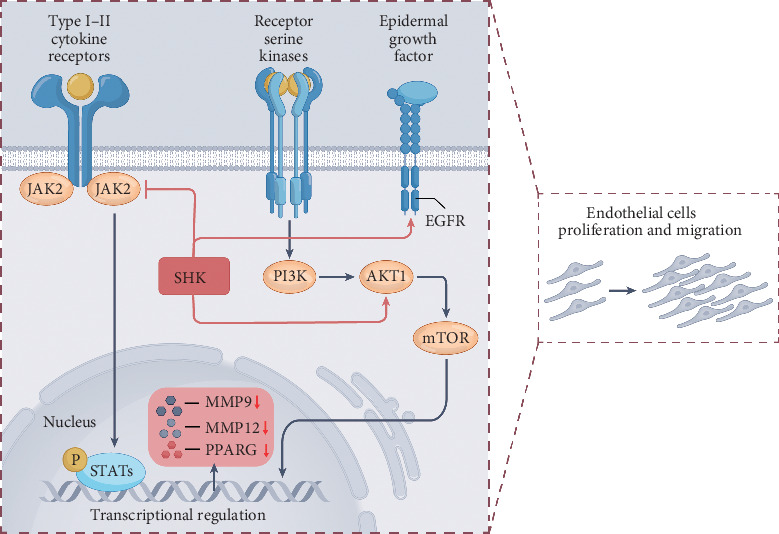
Schematic presentation of the PI3K–AKT signal transduction induced by SHK in HUVEC cells.

In summary, this study identified potential targets of action and biological pathways of SHK for the treatment of DW. SHK may regulate the PI3K–AKT signaling pathway by affecting EGFR, JAK2, and AKT1 to promote DW healing. In vitro experiments demonstrated that SHK could activate the PI3K–AKT signaling pathway and contribute to cell proliferation and migration (Figure 7).

## 5. Conclusions

This study revealed action of SHK on DW through multitarget and pathway interactions, highlighting the PI3K–AKT, MAPK, and AGE-RAGE pathways as central to its effects. Notably, SHK enhanced angiogenesis via the PI3K–AKT pathway, a key to its therapeutic benefit. This research underscored SHK's potential in DW treatment and the complexity of such conditions, advocating for an approach that integrated the interplay of diverse signaling pathways.

## Figures and Tables

**Table 1:** The binding energy of SHK with EGFR, JAK2, and AKT1.

**Component**	**Target**	**PDB ID**	**Blind energy (kcal/mol)**
Shikonin	EGFR	7tvd	−6.45
	JAK2	2b7a	−5.55
	AKT1	1unq	−5.81

## Data Availability

The data that support the findings of this study are available from the corresponding author upon reasonable request.

## Supporting Information

Supporting Information Additional supporting information can be found online in the Supporting Information section. Table S1: Primer sequences used for quantitative qRT-PCR. Table S2: Targets for shikonin from Pharmmapper database. Table S3: Targets for shikonin from Comparative Toxicogenomics Database. Table S4: Consolidated targets for diabetic wounds from GeneCards, OMIM, DisGeNET, Drugbank, and TTD databases. Table S5: Detailed KEGG and GO enrichment analysis results for SHK and DW intersecting genes.
