# Supplementary material for: Revealing the Mechanisms of Shikonin Against Diabetic Wounds: A Combined Network Pharmacology and In Vitro Investigation
Source: J Diabetes Res. 2025 Mar 10;2025:4656485. doi: 10.1155/jdr/4656485 (PMC11986939; doi:10.1155/jdr/4656485)
Supplement: Supporting Information — Additional supporting information can be found online in the Supporting Information section. Table S1: Primer sequences used for quantitative qRT-PCR. Table S2: Targets for shikonin from Pharmmapper database. Table S3: Targets for shikonin from Comparative Toxicogenomics Database. Table S4: Consolidated targets for diabetic wounds from GeneCards, OMIM, DisGeNET, Drugbank, and TTD databases. Table S5: Detailed KEGG and GO enrichment analysis results for SHK and DW intersecting genes. [file 4656485.f1.zip › Supplementary Table S1 Primer sequences used for quantitative qRT-PCR.docx]

**Table S1. Primer sequences used for quantitative qRT-PCR**

| **Gene** | **Primer sequence** |
| --- | --- |
| *PI3K* | Forward: 5’-CAT CAC TTC CTC CTG CTC TAT-3’ |
| *PI3K* | Reverse: 5’-CAG TTG TTG GCA ATC TTC TTC-3’ |
| *AKT1* | Forward: 5’-GAA GGA CGG GAG CAG GCG GC-3’ |
| *AKT1* | Reverse: 5’-CCT CCT CCA GGC AGC CCC TT-3’ |
| *EGFR* | Forward: 5’-GGC ACT TTT GAA GAT CAT TTT CTC-3’ |
| *EGFR* | Reverse: 5’-CTG TGT TGA GGG CAA TGA G-3’ |
| *JAK2* | Forward: 5’-GTC ATG GCC CAA TTT CGA T-3’ |
| *JAK2* | Reverse: 5’-TTC TTT GTC CCA CTG AGG TT-3’ |
| *GAPDH* | Forward: 5’-GTG GTC TCC TCT GAC TTC AAC A-3’ |
| *GAPDH* | Reverse: 5’-CTC TTC CTC TTG TGC TCT TGC T-3’ |
