# Supplementary material for: Revealing the Mechanisms of Shikonin Against Diabetic Wounds: A Combined Network Pharmacology and In Vitro Investigation
Source: J Diabetes Res. 2025 Mar 10;2025:4656485. doi: 10.1155/jdr/4656485 (PMC11986939; doi:10.1155/jdr/4656485)
Supplement: Supporting Information — Additional supporting information can be found online in the Supporting Information section. Table S1: Primer sequences used for quantitative qRT-PCR. Table S2: Targets for shikonin from Pharmmapper database. Table S3: Targets for shikonin from Comparative Toxicogenomics Database. Table S4: Consolidated targets for diabetic wounds from GeneCards, OMIM, DisGeNET, Drugbank, and TTD databases. Table S5: Detailed KEGG and GO enrichment analysis results for SHK and DW intersecting genes. [file 4656485.f1.zip › Supplementary Table S2 Targets for shikonin from Pharmmapper database.docx]

**Table S2. Targets for shikonin from Pharmmapper database**

| **Serial number** | **Targets of shikonin** |
| --- | --- |
| 1 | MMP3 |
| 2 | PDE5A |
| 3 | ESR1 |
| 4 | BMP2 |
| 5 | GSTP1 |
| 6 | APOA2 |
| 7 | KIF11 |
| 8 | EGFR |
| 9 | PDE4D |
| 10 | PDPK1 |
| 11 | MMP13 |
| 12 | PPARG |
| 13 | SHBG |
| 14 | ANXA5 |
| 15 | SRC |
| 16 | FGFR1 |
| 17 | DPP4 |
| 18 | NQO1 |
| 19 | EPHB4 |
| 20 | NOS3 |
| 21 | MMP8 |
| 22 | MIF |
| 23 | AKT1 |
| 24 | MET |
| 25 | NR1H3 |
| 26 | NR1H2 |
| 27 | MMP12 |
| 28 | NR1H4 |
| 29 | CASP3 |
| 30 | ERBB4 |
| 31 | JAK2 |
| 32 | MMP7 |
| 33 | AKT2 |
| 34 | IGF1R |
| 35 | NR1I2 |
| 36 | FABP4 |
| 37 | PPARA |
| 38 | IL2 |
| 39 | CCL5 |
| 40 | ACE |
| 41 | NOS2 |
| 42 | ALDH2 |
| 43 | MMP2 |
| 44 | TGFB2 |
| 45 | CASP1 |
| 46 | MMP9 |
| 47 | STAT1 |
| 48 | KIT |
| 49 | VDR |
| 50 | C1S |
| 51 | HMOX1 |
| 52 | HINT1 |
| 53 | CDC42 |
| 54 | DUT |
| 55 | INSR |
| 56 | GSTM1 |
| 57 | RAF1 |
| 58 | BTK |
| 59 | RAC1 |
| 60 | SIRT5 |
| 61 | CAH2 |
| 62 | EST1 |
| 63 | MK01 |
| 64 | CFAB |
| 65 | CHLE |
| 66 | THRB |
| 67 | BACE1 |
| 68 | VTDB |
| 69 | CMA1 |
| 70 | ANDR |
| 71 | LKHA4 |
| 72 | MK10 |
| 73 | CAH1 |
| 74 | TTHY |
| 75 | STS |
| 76 | MK08 |
| 77 | HCK |
| 78 | ADA17 |
| 79 | CD5R1 |
| 80 | ALBU |
| 81 | AOFB |
| 82 | ALDR |
| 83 | GLCM |
| 84 | CCNA2 |
| 85 | PDE4B |
| 86 | QPCT |
| 87 | CASP7 |
| 88 | CATL2 |
| 89 | CLPP |
| 90 | CHK1 |
| 91 | DHI1 |
| 92 | CBR1 |
| 93 | BRAF1 |
| 94 | MCR |
| 95 | RORA |
| 96 | PTN1 |
| 97 | CATB |
| 98 | RXRA |
| 99 | TYPH |
| 100 | CFAD |
| 101 | DAPK1 |
| 102 | PNMT |
| 103 | PRGR |
| 104 | AMPM2 |
| 105 | PDE3B |
| 106 | HS90A |
| 107 | ADH1B |
| 108 | FA10 |
| 109 | ANGI |
| 110 | CATK |
| 111 | PH4H |
| 112 | AK1C3 |
| 113 | PTN11 |
| 114 | PYRD |
| 115 | FNTA |
| 116 | PAK7 |
| 117 | TGFR1 |
| 118 | WASP |
| 119 | PTGD2 |
| 120 | CTNA1 |
| 121 | LCK |
| 122 | TYSY |
| 123 | DUS6 |
| 124 | ADHX |
| 125 | DYR |
| 126 | KC1G2 |
| 127 | ISG20 |
| 128 | DCK |
| 129 | RENI |
| 130 | FA7 |
| 131 | HMDH |
| 132 | ELNE |
| 133 | UROK |
| 134 | HYES |
| 135 | CDK6 |
| 136 | PLGF |
| 137 | DHSO |
| 138 | PADI4 |
| 139 | IMDH2 |
| 140 | PNPH |
| 141 | CDD |
| 142 | ADK |
| 143 | CP2C9 |
| 144 | ERG7 |
| 145 | PK3CG |
| 146 | CATG |
| 147 | BIRC7 |
| 148 | A1AT |
| 149 | KSYK |
| 150 | JAK3 |
| 151 | SAHH |
| 152 | FABP7 |
| 153 | AK1C2 |
| 154 | FKB1A |
| 155 | ZAP70 |
| 156 | CCNT1 |
| 157 | MDM2 |
| 158 | ACK1 |
| 159 | CATS |
| 160 | AMYP |
| 161 | NCS1 |
| 162 | XIAP |
| 163 | ST1E1 |
| 164 | ARSA |
| 165 | GLYC |
| 166 | NR1I3 |
| 167 | PDK2 |
| 168 | RET4 |
| 169 | BST1 |
| 170 | DPEP1 |
| 171 | PPAP |
| 172 | RAB5A |
| 173 | RB11A |
| 174 | PAK6 |
| 175 | TPIS |
| 176 | HXK1 |
| 177 | TGM3 |
| 178 | ST14 |
| 179 | ALDOA |
| 180 | CSK |
| 181 | NEP |
| 182 | KAT1 |
| 183 | SPYA |
| 184 | FABP6 |
| 185 | B3GA1 |
| 186 | HXK4 |
| 187 | CP2C8 |
| 188 | FABPH |
| 189 | TIE2 |
| 190 | NQO2 |
| 191 | LEG2 |
| 192 | CD1A |
| 193 | ITAL |
| 194 | G6PI |
| 195 | KPCT |
| 196 | GCR |
| 197 | GSTT2 |
| 198 | RARA |
| 199 | CLK1 |
| 200 | S10A9 |
| 201 | ARGI1 |
| 202 | SETD7 |
| 203 | KTHY |
| 204 | RARG |
| 205 | ACADM |
| 206 | TPH1 |
| 207 | LEG3 |
| 208 | MP2K1 |
| 209 | NGAL |
| 210 | EPHA2 |
| 211 | FA11 |
| 212 | IMPA1 |
| 213 | IMDH1 |
| 214 | PIMT |
| 215 | HEM2 |
| 216 | RASH |
| 217 | P85A |
| 218 | FPPS |
| 219 | TRYB2 |
| 220 | CD209 |
| 221 | OAT |
| 222 | FOLH1 |
| 223 | GSTO1 |
| 224 | GMPR1 |
| 225 | PUR2 |
| 226 | MMP16 |
| 227 | RARB |
| 228 | KPYR |
| 229 | FKB1B |
| 230 | PUR9 |
| 231 | LGUL |
| 232 | FIBG |
| 233 | FKBP3 |
| 234 | GSTM2 |
| 235 | GRB2 |
| 236 | GALE |
| 237 | ARGI2 |
| 238 | MK12 |
| 239 | LYAM2 |
| 240 | NDKB |
| 241 | RXRB |
| 242 | ADA33 |
| 243 | RAN |
| 244 | APAF |
| 245 | EPCR |
| 246 | MAOM |
| 247 | SETD8 |
| 248 | ST1A1 |
| 249 | AOFA |
