# Supplementary material for: Revealing the Mechanisms of Shikonin Against Diabetic Wounds: A Combined Network Pharmacology and In Vitro Investigation
Source: J Diabetes Res. 2025 Mar 10;2025:4656485. doi: 10.1155/jdr/4656485 (PMC11986939; doi:10.1155/jdr/4656485)
Supplement: Supporting Information — Additional supporting information can be found online in the Supporting Information section. Table S1: Primer sequences used for quantitative qRT-PCR. Table S2: Targets for shikonin from Pharmmapper database. Table S3: Targets for shikonin from Comparative Toxicogenomics Database. Table S4: Consolidated targets for diabetic wounds from GeneCards, OMIM, DisGeNET, Drugbank, and TTD databases. Table S5: Detailed KEGG and GO enrichment analysis results for SHK and DW intersecting genes. [file 4656485.f1.zip › Supplementary Table S3 Targets for shikonin from Comparative Toxicogenomics database.docx]

**Table S3.** **Targets for shikonin from Comparative Toxicogenomics database**

| **Serial number** | **Targets of shikonin** |
| --- | --- |
| 1 | ATF3 |
| 2 | EIF2AK3 |
| 3 | BCL2 |
| 4 | CASP3 |
| 5 | EIF2S1 |
| 6 | MAPK1 |
| 7 | MAPK3 |
| 8 | TNF |
| 9 | BAX |
| 10 | GPX4 |
| 11 | PARP1 |
| 12 | SLC7A11 |
| 13 | AKT1 |
| 14 | AXUD1 |
| 15 | CASP12 |
| 16 | CASP9 |
| 17 | CCL4 |
| 18 | CCL7 |
| 19 | CCND1 |
| 20 | CDH1 |
| 21 | CDH2 |
| 22 | CDKN1A |
| 23 | CYP1A2 |
| 24 | CYP2E1 |
| 25 | CYP3A2 |
| 26 | DDIT3 |
| 27 | DUSP1 |
| 28 | EGR2 |
| 29 | EGR3 |
| 30 | FOS |
| 31 | FOSB |
| 32 | HDAC1 |
| 33 | HIF1A |
| 34 | IL13 |
| 35 | ITGB1 |
| 36 | MAP3K8 |
| 37 | MYC |
| 38 | NFKBID |
| 39 | NFKBIZ |
| 40 | NR4A1 |
| 41 | NR4A2 |
| 42 | NR4A3 |
| 43 | PTGS2 |
| 44 | TAGAP |
| 45 | UGT1A1 |
| 46 | XBP1 |
| 47 | ZFP36 |
| 48 | ACTA2 |
| 49 | AHR |
| 50 | AREG |
| 51 | ATF6 |
| 52 | CASP7 |
| 53 | CCL1 |
| 54 | CCNE1 |
| 55 | COL1A1 |
| 56 | COL3A1 |
| 57 | CSF1 |
| 58 | CXCL1 |
| 59 | CYP2B1 |
| 60 | CYP2B6 |
| 61 | CYP2C11 |
| 62 | CYP2C9 |
| 63 | CYP2D1 |
| 64 | CYP2D6 |
| 65 | CYP3A4 |
| 66 | DUSP6 |
| 67 | EIF4EBP1 |
| 68 | ERN1 |
| 69 | HSF1 |
| 70 | IL1B |
| 71 | JUN |
| 72 | MAPK8 |
| 73 | MAPK9 |
| 74 | MMP2 |
| 75 | MMP3 |
| 76 | MMP7 |
| 77 | MMP9 |
| 78 | MT2 |
| 79 | MTOR |
| 80 | NFE2L2 |
| 81 | NKD2 |
| 82 | OLR1 |
| 83 | PCNA |
| 84 | PKM |
| 85 | PLK3 |
| 86 | PPP1R15A |
| 87 | RELA |
| 88 | RIPK1 |
| 89 | RIPK3 |
| 90 | RPS6KB1 |
| 91 | SERPINE1 |
| 92 | SNAI1 |
| 93 | SNAI2 |
| 94 | TAGAP1 |
| 95 | TIMP2 |
| 96 | TP53 |
| 97 | UGT1A3 |
| 98 | UGT1A9 |
| 99 | UGT2B7 |
| 100 | ZFP36L1 |
