# Supplementary material for: Revealing the Mechanisms of Shikonin Against Diabetic Wounds: A Combined Network Pharmacology and In Vitro Investigation
Source: J Diabetes Res. 2025 Mar 10;2025:4656485. doi: 10.1155/jdr/4656485 (PMC11986939; doi:10.1155/jdr/4656485)
Supplement: Supporting Information — Additional supporting information can be found online in the Supporting Information section. Table S1: Primer sequences used for quantitative qRT-PCR. Table S2: Targets for shikonin from Pharmmapper database. Table S3: Targets for shikonin from Comparative Toxicogenomics Database. Table S4: Consolidated targets for diabetic wounds from GeneCards, OMIM, DisGeNET, Drugbank, and TTD databases. Table S5: Detailed KEGG and GO enrichment analysis results for SHK and DW intersecting genes. [file 4656485.f1.zip › Supplementary Table S4 Consolidated targets for Diabetic Wounds from GeneCards, OMIM, DisGeNET, Drugbank, and TTD databases.docx]

**Table S4.** **Consolidated targets for** **Diabetic Wounds from GeneCards, OMIM, DisGeNET, Drugbank, and TTD databases**

| **Serial number** | **Targets of diabetic wounds** |
| --- | --- |
| 1 | MODY13 |
| 2 | IMD41 |
| 3 | T2D |
| 4 | COLED |
| 5 | PNDM2 |
| 6 | CTRCT41 |
| 7 | IDDM10 |
| 8 | IDDM22 |
| 9 | LYP |
| 10 | MEDS2 |
| 11 | MODY8 |
| 12 | PNDM4 |
| 13 | SUR1 |
| 14 | BSF2 |
| 15 | ESA |
| 16 | FKLF1 |
| 17 | HFE1 |
| 18 | HNF2 |
| 19 | MODY1 |
| 20 | MODY14 |
| 21 | MODY3 |
| 22 | MODY4 |
| 23 | P58 |
| 24 | PTPN8 |
| 25 | WFS |
| 26 | YIP1A |
| 27 | CD25 |
| 28 | IDDM5 |
| 29 | MODY9 |
| 30 | MVCD1 |
| 31 | MVCD4 |
| 32 | PDNP1 |
| 33 | TCF2 |
| 34 | TIEG2 |
| 35 | ZNF515 |
| 36 | ZNT8 |
| 37 | AVP |
| 38 | DNAJC3 |
| 39 | GCK |
| 40 | GLIS3 |
| 41 | HNF4A |
| 42 | IDDM19 |
| 43 | IDDM21 |
| 44 | INSR |
| 45 | ITPR3 |
| 46 | KCNJ11 |
| 47 | KLF11 |
| 48 | MAFA |
| 49 | MAPK8IP1 |
| 50 | PDX1 |
| 51 | PON1 |
| 52 | SLC30A8 |
| 53 | SOD2 |
| 54 | UCP3 |
| 55 | VEGF |
| 56 | WFS1 |
| 57 | PTPN1 |
| 58 | ADIPOQ |
| 59 | TGFB1 |
| 60 | IL1B |
| 61 | ADTKD3 |
| 62 | ARHR2 |
| 63 | DFNA38 |
| 64 | TNDM2 |
| 65 | DFNA14 |
| 66 | GLM1 |
| 67 | HGF |
| 68 | HHF1 |
| 69 | ICH |
| 70 | MODY7 |
| 71 | NDI1 |
| 72 | ODCD2 |
| 73 | PCA1 |
| 74 | TCP |
| 75 | TFQTL2 |
| 76 | TNDM3 |
| 77 | PNDM3 |
| 78 | WFSL |
| 79 | ACPHD |
| 80 | ADHR |
| 81 | ALPS5 |
| 82 | CIMT1 |
| 83 | DBAL |
| 84 | DFNA6 |
| 85 | FKLF |
| 86 | FRTS4 |
| 87 | HDLCQ12 |
| 88 | HHF2 |
| 89 | HSF |
| 90 | IDDM20 |
| 91 | KIAA0268 |
| 92 | M6S1 |
| 93 | MVCD3 |
| 94 | MVCD5 |
| 95 | MVCD7 |
| 96 | NIDDM5 |
| 97 | PAGEN1 |
| 98 | RCAD |
| 99 | TATI |
| 100 | ACE1 |
| 101 | CCCKR5 |
| 102 | CELIAC3 |
| 103 | CELL |
| 104 | DI1 |
| 105 | DIRA |
| 106 | ECYT5 |
| 107 | FIZZ3 |
| 108 | IDDM2 |
| 109 | IL2R |
| 110 | INSDM |
| 111 | KIAA0603 |
| 112 | KPD |
| 113 | LIPH |
| 114 | MFH1 |
| 115 | MVCD6 |
| 116 | NDH |
| 117 | NPPS |
| 118 | PCTT |
| 119 | PHHI |
| 120 | PNDM1 |
| 121 | PPARG2 |
| 122 | TANGO |
| 123 | VP |
| 124 | APPL |
| 125 | AS160 |
| 126 | AVRP |
| 127 | BIR |
| 128 | BMFDMS |
| 129 | BSSL |
| 130 | CMKBR5 |
| 131 | CMT1J |
| 132 | DCP1 |
| 133 | DIR |
| 134 | FKHL14 |
| 135 | GLUT2 |
| 136 | HHF3 |
| 137 | HHF5 |
| 138 | HIHGHH |
| 139 | HL |
| 140 | HLA-H |
| 141 | HMGIY |
| 142 | IB1 |
| 143 | IDDM12 |
| 144 | IFNB2 |
| 145 | IMP2 |
| 146 | IPF1 |
| 147 | MEDS |
| 148 | MNSOD |
| 149 | MODY10 |
| 150 | MODY11 |
| 151 | MVCD2 |
| 152 | NDI2 |
| 153 | NIDDM1 |
| 154 | PEP |
| 155 | PON |
| 156 | PPARG1 |
| 157 | PRKRI |
| 158 | PSTI |
| 159 | RIPE3B1 |
| 160 | RSTN |
| 161 | SMAP5 |
| 162 | SUR |
| 163 | TANGO1 |
| 164 | TCF1 |
| 165 | TCF14 |
| 166 | TCF4 |
| 167 | TNDM1 |
| 168 | WFRS |
| 169 | ABCC8 |
| 170 | ACE |
| 171 | AKT2 |
| 172 | APPL1 |
| 173 | AQP2 |
| 174 | AVPR2 |
| 175 | BLK |
| 176 | CAPN10 |
| 177 | CCR5 |
| 178 | CEL |
| 179 | CTLA4 |
| 180 | DUT |
| 181 | ENPP1 |
| 182 | EPO |
| 183 | FOXC2 |
| 184 | GPD2 |
| 185 | HFE |
| 186 | HMGA1 |
| 187 | HNF1A |
| 188 | HNF1B |
| 189 | IAPP |
| 190 | IDDM1 |
| 191 | IDDM11 |
| 192 | IDDM13 |
| 193 | IDDM15 |
| 194 | IDDM17 |
| 195 | IDDM18 |
| 196 | IDDM23 |
| 197 | IDDM24 |
| 198 | IDDM3 |
| 199 | IDDM4 |
| 200 | IDDM6 |
| 201 | IDDM7 |
| 202 | IDDM8 |
| 203 | IDDMX |
| 204 | IER3IP1 |
| 205 | IGF2BP2 |
| 206 | IL1RN |
| 207 | IL2RA |
| 208 | IL6 |
| 209 | INS |
| 210 | IRS1 |
| 211 | IRS2 |
| 212 | LIPC |
| 213 | MIA3 |
| 214 | MTNR1B |
| 215 | NEUROD1 |
| 216 | NIDDM2 |
| 217 | NIDDM4 |
| 218 | PAX4 |
| 219 | PBCA |
| 220 | PPARG |
| 221 | PTPN22 |
| 222 | RETN |
| 223 | SLC2A2 |
| 224 | SPINK1 |
| 225 | SUMO4 |
| 226 | T2D3 |
| 227 | TBC1D4 |
| 228 | TCF7L2 |
| 229 | TNDM1 |
| 230 | WAKMAR1 |
| 231 | WAKMAR2 |
| 232 | YIPF5 |
| 233 | ZFP57 |
| 234 | VEGFA |
| 235 | LMNA |
| 236 | INS-IGF2 |
| 237 | ALB |
| 238 | TNF |
| 239 | MIR29A |
| 240 | SERPINE1 |
| 241 | IGF1 |
| 242 | MIR21 |
| 243 | LEP |
| 244 | MIR29C |
| 245 | GCG |
| 246 | CRP |
| 247 | AGER |
| 248 | CCL2 |
| 249 | AKR1B1 |
| 250 | STAT3 |
| 251 | HLA-DRB1 |
| 252 | MIR17 |
| 253 | CAT |
| 254 | APOE |
| 255 | NOS3 |
| 256 | CXCL8 |
| 257 | MIR155 |
| 258 | MMP9 |
| 259 | COL1A1 |
| 260 | ICAM1 |
| 261 | AKT1 |
| 262 | IL10 |
| 263 | MTHFR |
| 264 | TP53 |
| 265 | FGF2 |
| 266 | DPP4 |
| 267 | FOXP3 |
| 268 | PIK3R1 |
| 269 | APOB |
| 270 | MEN1 |
| 271 | SLC2A1 |
| 272 | LPL |
| 273 | MT-ND1 |
| 274 | MIR145 |
| 275 | CDKN2A |
| 276 | GHRL |
| 277 | NGF |
| 278 | CISD2 |
| 279 | CTNNB1 |
| 280 | FN1 |
| 281 | CP |
| 282 | LEPR |
| 283 | FBN1 |
| 284 | APOA1 |
| 285 | GNAS |
| 286 | COL1A2 |
| 287 | ADRB3 |
| 288 | HFE-AS1 |
| 289 | ELN |
| 290 | VWF |
| 291 | EDN1 |
| 292 | MIR140 |
| 293 | TLR4 |
| 294 | GATA6 |
| 295 | LIPE |
| 296 | F13A1 |
| 297 | MMP1 |
| 298 | ADRB2 |
| 299 | CAV1 |
| 300 | MIR192 |
| 301 | ATM |
| 302 | POMC |
| 303 | CD36 |
| 304 | IFNG |
| 305 | AGTR1 |
| 306 | FTO |
| 307 | AGT |
| 308 | RRAD |
| 309 | EGF |
| 310 | IL4 |
| 311 | HMOX1 |
| 312 | PPARA |
| 313 | REN |
| 314 | BDNF |
| 315 | VCAM1 |
| 316 | SOD1 |
| 317 | PLIN1 |
| 318 | SELE |
| 319 | CCN2 |
| 320 | STAT1 |
| 321 | MMP2 |
| 322 | IGF1R |
| 323 | PIK3CA |
| 324 | AEBP1 |
| 325 | IFIH1 |
| 326 | HHEX |
| 327 | IL18 |
| 328 | HIF1A |
| 329 | FLT1 |
| 330 | BMP2 |
| 331 | MIR30E |
| 332 | IL2 |
| 333 | IGFBP1 |
| 334 | KCNQ1 |
| 335 | RBP4 |
| 336 | GJA1 |
| 337 | NAMPT |
| 338 | SST |
| 339 | FGFR1 |
| 340 | AOC3 |
| 341 | PCSK1 |
| 342 | IGF2 |
| 343 | THBD |
| 344 | GHR |
| 345 | IGFBP3 |
| 346 | PTEN |
| 347 | CFTR |
| 348 | PPARGC1A |
| 349 | TH |
| 350 | SELP |
| 351 | MAPK1 |
| 352 | PDGFB |
| 353 | SIRT1 |
| 354 | FOS |
| 355 | LPA |
| 356 | NOS2 |
| 357 | MIR132 |
| 358 | HBA1 |
| 359 | VDR |
| 360 | MIR126 |
| 361 | PLAT |
| 362 | ITGB2 |
| 363 | CPE |
| 364 | IDE |
| 365 | CDKN2B |
| 366 | PRKCB |
| 367 | AIP |
| 368 | WRN |
| 369 | F2 |
| 370 | PCBD1 |
| 371 | PLG |
| 372 | MLXIPL |
| 373 | F8 |
| 374 | PGF |
| 375 | GPT |
| 376 | CETP |
| 377 | HAMP |
| 378 | HGF |
| 379 | IL1A |
| 380 | CASR |
| 381 | PNPLA2 |
| 382 | LTA |
| 383 | IL17A |
| 384 | F13B |
| 385 | MIR143 |
| 386 | MIR146A |
| 387 | MIR34A |
| 388 | MTOR |
| 389 | MIR375 |
| 390 | GH1 |
| 391 | TIMP1 |
| 392 | MIR20A |
| 393 | MIF |
| 394 | NPY |
| 395 | CXCL10 |
| 396 | MAPK8 |
| 397 | SLC40A1 |
| 398 | SLC12A3 |
| 399 | GSK3B |
| 400 | BGLAP |
| 401 | KDR |
| 402 | CASP3 |
| 403 | ACP1 |
| 404 | CST3 |
| 405 | NFKB1 |
| 406 | ADM |
| 407 | HLA-B |
| 408 | HP |
| 409 | MIR99A |
| 410 | PIK3CG |
| 411 | MIR122 |
| 412 | GGT1 |
| 413 | TTR |
| 414 | BCL2 |
| 415 | MAPK14 |
| 416 | HLA-A |
| 417 | NFE2L2 |
| 418 | IL13 |
| 419 | LAMA3 |
| 420 | TLR2 |
| 421 | PTGS2 |
| 422 | HJV |
| 423 | NPPA |
| 424 | ABCA1 |
| 425 | SMAD3 |
| 426 | CDKN3 |
| 427 | TGIF1 |
| 428 | UCP1 |
| 429 | NOTCH2 |
| 430 | PPARD |
| 431 | APOA5 |
| 432 | BMP6 |
| 433 | MIR221 |
| 434 | HMGCR |
| 435 | ST3GAL4 |
| 436 | MIR483 |
| 437 | G6PC1 |
| 438 | MIR377 |
| 439 | PTPRN2 |
| 440 | NLRP3 |
| 441 | TFR2 |
| 442 | PRL |
| 443 | TGFBR2 |
| 444 | USP8 |
| 445 | TNXB |
| 446 | MEG3 |
| 447 | STK11 |
| 448 | COL5A1 |
| 449 | HMGB1 |
| 450 | PLA2G6 |
| 451 | FOXO1 |
| 452 | CXCL12 |
| 453 | SOCS3 |
| 454 | FGF21 |
| 455 | FGF7 |
| 456 | CLCNKB |
| 457 | GIP |
| 458 | TGFBR1 |
| 459 | LEPQTL1 |
| 460 | NTRK1 |
| 461 | ACE2 |
| 462 | IGFBP2 |
| 463 | EGFR |
| 464 | FABP2 |
| 465 | PEA15 |
| 466 | G6PC2 |
| 467 | MIR197 |
| 468 | ANGPT2 |
| 469 | AMBP |
| 470 | AR |
| 471 | ADIPOR2 |
| 472 | F9 |
| 473 | MIR27A |
| 474 | IL6R |
| 475 | PRKAA2 |
| 476 | FOXA2 |
| 477 | MIR134 |
| 478 | MT-ND4 |
| 479 | SERPINF1 |
| 480 | ESR1 |
| 481 | TGM2 |
| 482 | PVT1 |
| 483 | RAC2 |
| 484 | FABP4 |
| 485 | APLN |
| 486 | SRC |
| 487 | SELL |
| 488 | CNR1 |
| 489 | TGFB2 |
| 490 | GSR |
| 491 | LCN2 |
| 492 | NPPB |
| 493 | TKT |
| 494 | SOD3 |
| 495 | AHSG |
| 496 | NR3C1 |
| 497 | POLG |
| 498 | CALCA |
| 499 | ANGPTL4 |
| 500 | XBP1 |
| 501 | CRYAA |
| 502 | GAPDH |
| 503 | MMP3 |
| 504 | F3 |
| 505 | MIR223 |
| 506 | TNFRSF1B |
| 507 | PRKCA |
| 508 | PRKAA1 |
| 509 | SREBF1 |
| 510 | HSPA4 |
| 511 | MPO |
| 512 | CD4 |
| 513 | SGK1 |
| 514 | CYP3A4 |
| 515 | PIK3C2A |
| 516 | NPHS1 |
| 517 | SERPINC1 |
| 518 | TAC1 |
| 519 | HSPA5 |
| 520 | STX1A |
| 521 | CACNA1A |
| 522 | SLC17A5 |
| 523 | BRAF |
| 524 | SLC2A3 |
| 525 | CREB1 |
| 526 | F5 |
| 527 | CYP19A1 |
| 528 | BBS2 |
| 529 | HSD11B1 |
| 530 | NR1H2 |
| 531 | TF |
| 532 | F7 |
| 533 | ELANE |
| 534 | C3 |
| 535 | JUN |
| 536 | VTN |
| 537 | SOX2 |
| 538 | THBS1 |
| 539 | MIR210 |
| 540 | SERPINF2 |
| 541 | SHH |
| 542 | CCR6 |
| 543 | AGTR2 |
| 544 | GFPT1 |
| 545 | MIR181A1 |
| 546 | DLK1 |
| 547 | PLA2G7 |
| 548 | ACHE |
| 549 | KCNJ5 |
| 550 | SCARB1 |
| 551 | FNDC5 |
| 552 | CYBA |
| 553 | SCN11A |
| 554 | TNFRSF11B |
| 555 | CCL5 |
| 556 | PCSK9 |
| 557 | ALDH2 |
| 558 | SOCS1 |
| 559 | MT-ND5 |
| 560 | PNPLA3 |
| 561 | IGFBP7 |
| 562 | HSPD1 |
| 563 | GIPR |
| 564 | CDH23 |
| 565 | CDKN2B-AS1 |
| 566 | TGFB3 |
| 567 | IL15 |
| 568 | RPS6KB1 |
| 569 | SERPINA3 |
| 570 | MIR93 |
| 571 | CYP21A2 |
| 572 | LCAT |
| 573 | SPP1 |
| 574 | CYP2E1 |
| 575 | APP |
| 576 | IL1R1 |
| 577 | FADS1 |
| 578 | G6PD |
| 579 | MT-CO2 |
| 580 | ERN1 |
| 581 | MT-CO1 |
| 582 | COL5A2 |
| 583 | NOX4 |
| 584 | GAL |
| 585 | CFH |
| 586 | ARL6 |
| 587 | BRCA2 |
| 588 | MAP2K1 |
| 589 | MIR23A |
| 590 | PRKCZ |
| 591 | MAPK3 |
| 592 | PIK3CB |
| 593 | B2M |
| 594 | TG |
| 595 | CASP1 |
| 596 | TSC1 |
| 597 | MIR22 |
| 598 | CCR2 |
| 599 | PAPPA |
| 600 | GC |
| 601 | TIMP2 |
| 602 | SMAD4 |
| 603 | TNFRSF1A |
| 604 | INPPL1 |
| 605 | PRSS1 |
| 606 | CACNA1H |
| 607 | RHOA |
| 608 | CD8A |
| 609 | HBEGF |
| 610 | TSPAN8 |
| 611 | ANGPT1 |
| 612 | ALOX12 |
| 613 | EP300 |
| 614 | CAPN5 |
| 615 | ADAMTS9 |
| 616 | OXT |
| 617 | LRP5 |
| 618 | CCK |
| 619 | MMP8 |
| 620 | CNTF |
| 621 | HBB |
| 622 | GSTM1 |
| 623 | RAC1 |
| 624 | CD38 |
| 625 | APOA2 |
| 626 | CSN1S1 |
| 627 | PTH |
| 628 | FOXO3 |
| 629 | MIR15A |
| 630 | CD40 |
| 631 | MIR18A |
| 632 | MT-ATP8 |
| 633 | SI |
| 634 | H2AC18 |
| 635 | BAIAP2L1 |
| 636 | COL7A1 |
| 637 | RHO |
| 638 | PTPN3 |
| 639 | GNAI2 |
| 640 | ONECUT1 |
| 641 | GLI2 |
| 642 | BCHE |
| 643 | NFKBIA |
| 644 | USF1 |
| 645 | PRKAB1 |
| 646 | SERPINA1 |
| 647 | HRAS |
| 648 | MIR106B |
| 649 | SCT |
| 650 | CTSB |
| 651 | CSF3 |
| 652 | CD44 |
| 653 | HK2 |
| 654 | APOC2 |
| 655 | MET |
| 656 | PRKAR1A |
| 657 | GAS5 |
| 658 | MALAT1 |
| 659 | CXCR4 |
| 660 | NAGLU |
| 661 | PNLIP |
| 662 | JAK2 |
| 663 | PARP1 |
| 664 | EXT2 |
| 665 | CYP17A1 |
| 666 | NCF1 |
| 667 | MMP13 |
| 668 | FASLG |
| 669 | SLC11A1 |
| 670 | MIR127 |
| 671 | MMP14 |
| 672 | MBL2 |
| 673 | LAMC2 |
| 674 | ITGB1 |
| 675 | CSF2 |
| 676 | SCAPER |
| 677 | EDNRA |
| 678 | SAG |
| 679 | HSP90AA1 |
| 680 | IKBKB |
| 681 | CD63 |
| 682 | PDE11A |
| 683 | FOXM1 |
| 684 | PDE8B |
| 685 | ENG |
| 686 | NTS |
| 687 | DDIT3 |
| 688 | ITGAM |
| 689 | NR3C2 |
| 690 | NOD2 |
| 691 | MAPK10 |
| 692 | HSD11B2 |
| 693 | ADCY10 |
| 694 | TGFA |
| 695 | MIR195 |
| 696 | BAZ1B |
| 697 | HSPA1A |
| 698 | VANGL1 |
| 699 | ABCA4 |
| 700 | CS |
| 701 | CCL11 |
| 702 | SHC1 |
| 703 | SCD |
| 704 | ACACA |
| 705 | PTCH1 |
| 706 | PECAM1 |
| 707 | AQP3 |
| 708 | DIO2 |
| 709 | MIR199A1 |
| 710 | FGF8 |
| 711 | GRN |
| 712 | GFAP |
| 713 | SIX3 |
| 714 | TBL2 |
| 715 | RPE65 |
| 716 | APC |
| 717 | CFI |
| 718 | MFN2 |
| 719 | ESR2 |
| 720 | PAX6 |
| 721 | PTK2B |
| 722 | COL17A1 |
| 723 | MIR423 |
| 724 | CPT1A |
| 725 | FASN |
| 726 | CYCS |
| 727 | PLIN2 |
| 728 | MIR142 |
| 729 | CD40LG |
| 730 | SLC9A1 |
| 731 | GPX1 |
| 732 | LOX |
| 733 | MIR224 |
| 734 | CPT2 |
| 735 | CD79A |
| 736 | CYBB |
| 737 | SNAP23 |
| 738 | LAMB3 |
| 739 | TTC8 |
| 740 | SAA1 |
| 741 | CYP2D6 |
| 742 | MB |
| 743 | FABP1 |
| 744 | ALOX5 |
| 745 | FAP |
| 746 | NGFR |
| 747 | MKKS |
| 748 | TRPV1 |
| 749 | PSMB8 |
| 750 | VPS33B |
| 751 | TIMP3 |
| 752 | PLCG1 |
| 753 | PTS |
| 754 | MIR486-1 |
| 755 | IGF2R |
| 756 | FST |
| 757 | CXCL1 |
| 758 | IL5 |
| 759 | CDH5 |
| 760 | FABP3 |
| 761 | LGALS3 |
| 762 | FGFR2 |
| 763 | SIRT3 |
| 764 | MIR182 |
| 765 | TERT |
| 766 | MIR200B |
| 767 | OPRM1 |
| 768 | SLC9A3 |
| 769 | ITGA2 |
| 770 | DLL1 |
| 771 | PRKACA |
| 772 | C4A |
| 773 | BMAL1 |
| 774 | OLR1 |
| 775 | B3GALT6 |
| 776 | GBA1 |
| 777 | MIR125A |
| 778 | PF4 |
| 779 | MIR296 |
| 780 | KRAS |
| 781 | FGA |
| 782 | PTX3 |
| 783 | TEK |
| 784 | SIRT6 |
| 785 | MST1 |
| 786 | MAPT |
| 787 | CYP1A2 |
| 788 | PRPS1 |
| 789 | PMM2 |
| 790 | MIR222 |
| 791 | SREBF2 |
| 792 | KRT5 |
| 793 | PRTN3 |
| 794 | CFAP418 |
| 795 | KL |
| 796 | DCN |
| 797 | DRD2 |
| 798 | SP1 |
| 799 | ITGAX |
| 800 | NTF3 |
| 801 | IFT172 |
| 802 | MIR9-1 |
| 803 | MIR335 |
| 804 | GAST |
| 805 | CEBPB |
| 806 | TCF4 |
| 807 | CCL3 |
| 808 | CCKAR |
| 809 | MIR149 |
| 810 | KNG1 |
| 811 | HTR2A |
| 812 | LIMK1 |
| 813 | ITGB3 |
| 814 | MIR144 |
| 815 | OCA2 |
| 816 | GATA4 |
| 817 | HPSE |
| 818 | TFAP2A |
| 819 | MIR130B |
| 820 | HSPA8 |
| 821 | NOS1 |
| 822 | LDLR |
| 823 | S100A9 |
| 824 | ESRRA |
| 825 | TDGF1 |
| 826 | PLA2G2A |
| 827 | ZIC2 |
| 828 | CD163 |
| 829 | MIR15B |
| 830 | MERTK |
| 831 | CD68 |
| 832 | ARG1 |
| 833 | SERPINA6 |
| 834 | USH2A |
| 835 | KRT14 |
| 836 | ANXA1 |
| 837 | TNC |
| 838 | PROX1 |
| 839 | IFNB1 |
| 840 | TLR5 |
| 841 | GPBAR1 |
| 842 | PWRN1 |
| 843 | GUSB |
| 844 | CISH |
| 845 | ITGB4 |
| 846 | MIR150 |
| 847 | CEBPA |
| 848 | PROM1 |
| 849 | FGF10 |
| 850 | FGF1 |
| 851 | EPB41L4B |
| 852 | MIR30A |
| 853 | GDNF |
| 854 | MIP |
| 855 | BMP7 |
| 856 | GSTP1 |
| 857 | ANPEP |
| 858 | CORIN |
| 859 | GPR35 |
| 860 | ERBB3 |
| 861 | MIR30B |
| 862 | BEST1 |
| 863 | TP63 |
| 864 | STAR |
| 865 | SFTA3 |
| 866 | CCN1 |
| 867 | TFAP2B |
| 868 | PLEK |
| 869 | TNFSF11 |
| 870 | CREBBP |
| 871 | PTPRC |
| 872 | CFB |
| 873 | FFAR4 |
| 874 | CHGA |
| 875 | ACTB |
| 876 | FABP12 |
| 877 | PRKCD |
| 878 | TLR9 |
| 879 | VEGFB |
| 880 | MYLK |
| 881 | CASP8 |
| 882 | CYP7A1 |
| 883 | MSTN |
| 884 | TYK2 |
| 885 | ATL1 |
| 886 | MIR455 |
| 887 | CHI3L1 |
| 888 | PTGDS |
| 889 | PLAU |
| 890 | C1R |
| 891 | PTPN2 |
| 892 | MIRLET7B |
| 893 | MIR342 |
| 894 | ADORA1 |
| 895 | STUB1 |
| 896 | IMPG2 |
| 897 | ERBB2 |
| 898 | BRCA1 |
| 899 | POSTN |
| 900 | KCTD1 |
| 901 | PDGFA |
| 902 | DUSP1 |
| 903 | MIR19B1 |
| 904 | GDF15 |
| 905 | MIR100 |
| 906 | MAP2K6 |
| 907 | ASAH1 |
| 908 | SIK2 |
| 909 | MIR196A1 |
| 910 | SLPI |
| 911 | TLR3 |
| 912 | UROD |
| 913 | MIR130A |
| 914 | AHR |
| 915 | EYS |
| 916 | MIR574 |
| 917 | SATB2 |
| 918 | COL3A1 |
| 919 | CD69 |
| 920 | ACSL1 |
| 921 | FGF23 |
| 922 | PLOD1 |
| 923 | AQP1 |
| 924 | COL6A1 |
| 925 | CD46 |
| 926 | MIR378A |
| 927 | CAMP |
| 928 | TFRC |
| 929 | DYRK1B |
| 930 | H19 |
| 931 | RBPJ |
| 932 | TULP1 |
| 933 | MIR135A1 |
| 934 | PRPH2 |
| 935 | CD14 |
| 936 | SIRT4 |
| 937 | MYD88 |
| 938 | NOTCH3 |
| 939 | CLU |
| 940 | PRDM16 |
| 941 | S100A8 |
| 942 | CD28 |
| 943 | PALLD |
| 944 | MIR96 |
| 945 | MMP7 |
| 946 | CRH |
| 947 | MIRLET7A1 |
| 948 | GJB2 |
| 949 | MIR136 |
| 950 | MIR33B |
| 951 | PLEC |
| 952 | CACNA1C |
| 953 | SIRT2 |
| 954 | PDPK1 |
| 955 | KCNJ1 |
| 956 | NOX1 |
| 957 | ADA2 |
| 958 | SMAD2 |
| 959 | MIR193B |
| 960 | MIR324 |
| 961 | IL12B |
| 962 | MACF1 |
| 963 | CD274 |
| 964 | TSC2 |
| 965 | S100B |
| 966 | KRT18 |
| 967 | BLOC1S1 |
| 968 | MIR214 |
| 969 | LAMA5 |
| 970 | DNMT3A |
| 971 | GSTT1 |
| 972 | AFG3L2 |
| 973 | MYOD1 |
| 974 | AHI1 |
| 975 | MIR206 |
| 976 | MIR532 |
| 977 | COMT |
| 978 | MIR34C |
| 979 | HMGA2 |
| 980 | SDC1 |
| 981 | MIR181A2 |
| 982 | MT-ND2 |
| 983 | MIR212 |
| 984 | SPARC |
| 985 | KRT6A |
| 986 | LRAT |
| 987 | IMPG1 |
| 988 | MIR193A |
| 989 | VIP |
| 990 | CXCR2 |
| 991 | BMP4 |
| 992 | AVPR1B |
| 993 | KDM4C |
| 994 | MIR148A |
| 995 | PDE6A |
| 996 | DST |
| 997 | PTGS1 |
| 998 | VEGFC |
| 999 | ANXA5 |
| 1000 | IGFBP4 |
| 1001 | NDUFS4 |
| 1002 | MIR590 |
| 1003 | RDH12 |
| 1004 | SCN9A |
| 1005 | TCF3 |
| 1006 | TJP1 |
| 1007 | MT-ND3 |
| 1008 | SEMA4D |
| 1009 | PDGFRB |
| 1010 | C2CD4A |
| 1011 | MIR675 |
| 1012 | UCHL1 |
| 1013 | ITGA2B |
| 1014 | FCGR2A |
| 1015 | FLNC |
| 1016 | RBP3 |
| 1017 | PTK2 |
| 1018 | SLC12A1 |
| 1019 | BTNL2 |
| 1020 | MIRLET7D |
| 1021 | SCARB2 |
| 1022 | CYP2C19 |
| 1023 | ITGA6 |
| 1024 | GATA3 |
| 1025 | SIRT7 |
| 1026 | NOTCH1 |
| 1027 | COL6A2 |
| 1028 | MIR203A |
| 1029 | PRPF31 |
| 1030 | IGF2-AS |
| 1031 | GNRH1 |
| 1032 | CSMD1 |
| 1033 | TOR1A |
| 1034 | PRPF8 |
| 1035 | CELF1 |
| 1036 | MIR19A |
| 1037 | MIR146B |
| 1038 | C1S |
| 1039 | BSND |
| 1040 | CD34 |
| 1041 | GATA1 |
| 1042 | PDGFRA |
| 1043 | ADRA2A |
| 1044 | ADA |
| 1045 | ADAM17 |
| 1046 | IFT88 |
| 1047 | CDH1 |
| 1048 | VIM |
| 1049 | CNBP |
| 1050 | SPTLC1 |
| 1051 | HLA-C |
| 1052 | ACVR1B |
| 1053 | KLF4 |
| 1054 | NRP1 |
| 1055 | RUNX2 |
| 1056 | PFKM |
| 1057 | WFDC21P |
| 1058 | RPGR |
| 1059 | LGR5 |
| 1060 | CDK5 |
| 1061 | OFD1 |
| 1062 | IL2RB |
| 1063 | XDH |
| 1064 | MMP10 |
| 1065 | MYC |
| 1066 | ROBO1 |
| 1067 | CYP11B2 |
| 1068 | AKR1B10 |
| 1069 | FGFR4 |
| 1070 | LZTFL1 |
| 1071 | PDE5A |
| 1072 | INHBA |
| 1073 | DGKB |
| 1074 | F2R |
| 1075 | GP1BA |
| 1076 | SPTLC2 |
| 1077 | LRP1 |
| 1078 | KCNQ1OT1 |
| 1079 | ZNF513 |
| 1080 | GZMB |
| 1081 | SMPD1 |
| 1082 | COL2A1 |
| 1083 | CERKL |
| 1084 | PLAUR |
| 1085 | SOST |
| 1086 | GAP43 |
| 1087 | CASP9 |
| 1088 | FAS |
| 1089 | SIRT5 |
| 1090 | MIR10B |
| 1091 | NQO1 |
| 1092 | PTGER4 |
| 1093 | POU5F1 |
| 1094 | CCL4 |
| 1095 | NKX2-5 |
| 1096 | MIR133A1 |
| 1097 | FGFR3 |
| 1098 | ELMO1 |
| 1099 | FBLN5 |
| 1100 | BRIP1 |
| 1101 | AGBL5 |
| 1102 | MYH9 |
| 1103 | ATXN2 |
| 1104 | ADAMTS13 |
| 1105 | MIRLET7I |
| 1106 | TUB |
| 1107 | PDE4D |
| 1108 | CTSD |
| 1109 | RAF1 |
| 1110 | LRP6 |
| 1111 | FTH1 |
| 1112 | STIL |
| 1113 | AFP |
| 1114 | TRH |
| 1115 | AQP4 |
| 1116 | ZNF408 |
| 1117 | CA4 |
| 1118 | IL7 |
| 1119 | PRKCE |
| 1120 | CDC42 |
| 1121 | HRC |
| 1122 | MIR188 |
| 1123 | MMP12 |
| 1124 | STAP2 |
| 1125 | CPB1 |
| 1126 | EPHB2 |
| 1127 | CDKN1A |
| 1128 | REEP6 |
| 1129 | PRRX2 |
| 1130 | IL7R |
| 1131 | ITCH |
| 1132 | MIR25 |
| 1133 | FGB |
| 1134 | C6orf89 |
| 1135 | LACTB |
| 1136 | SLC2A10 |
| 1137 | GHSR |
| 1138 | DEFB4A |
| 1139 | CXCR3 |
| 1140 | KLC2 |
| 1141 | MNX1 |
| 1142 | GPX3 |
| 1143 | DNMT1 |
| 1144 | MIR382 |
| 1145 | CAPN2 |
| 1146 | TMPRSS6 |
| 1147 | EREG |
| 1148 | MIR345 |
| 1149 | PDE6B |
| 1150 | CRB1 |
| 1151 | SERPINH1 |
| 1152 | ANGPTL3 |
| 1153 | MIR20B |
| 1154 | ANXA2 |
| 1155 | RP1L1 |
| 1156 | PRKCQ |
| 1157 | MT-TT |
| 1158 | PMP22 |
| 1159 | HSPB1 |
| 1160 | ROM1 |
| 1161 | CHST14 |
| 1162 | MIR181C |
| 1163 | CXCL9 |
| 1164 | KEAP1 |
| 1165 | RECK |
| 1166 | AQP5 |
| 1167 | AXL |
| 1168 | MIR181B1 |
| 1169 | MIR323A |
| 1170 | SPI1 |
| 1171 | SLC39A13 |
| 1172 | MIR204 |
| 1173 | CDK4 |
| 1174 | IMPDH1 |
| 1175 | SNAI2 |
| 1176 | ATL3 |
| 1177 | LDHA |
| 1178 | IFNA1 |
| 1179 | CACNA1D |
| 1180 | FCGR3B |
| 1181 | RLBP1 |
| 1182 | SLC29A1 |
| 1183 | GLDC |
| 1184 | CNGB1 |
| 1185 | KRT10 |
| 1186 | NUDC |
| 1187 | KCNN4 |
| 1188 | ABCB1 |
| 1189 | GREM1 |
| 1190 | ZFP36 |
| 1191 | COX5A |
| 1192 | CTNND1 |
| 1193 | CRY2 |
| 1194 | NUDT6 |
| 1195 | SCG5 |
| 1196 | DNM1L |
| 1197 | IGFBP5 |
| 1198 | ARHGEF18 |
| 1199 | DHDDS |
| 1200 | EGR1 |
| 1201 | DHX38 |
| 1202 | KLKB1 |
| 1203 | NR2E3 |
| 1204 | REG3A |
| 1205 | PKD2 |
| 1206 | CRX |
| 1207 | ITGA3 |
| 1208 | PLCD1 |
| 1209 | NRG1 |
| 1210 | SLC16A1 |
| 1211 | P2RY12 |
| 1212 | L1CAM |
| 1213 | NRAS |
| 1214 | PRPF6 |
| 1215 | LTF |
| 1216 | MCAM |
| 1217 | ARL3 |
| 1218 | NRL |
| 1219 | CCND1 |
| 1220 | GPHN |
| 1221 | VIPAS39 |
| 1222 | SCN10A |
| 1223 | IFT140 |
| 1224 | PTPN11 |
| 1225 | MAP3K5 |
| 1226 | ELAVL1 |
| 1227 | OCLN |
| 1228 | ITGB6 |
| 1229 | TYMP |
| 1230 | GP2 |
| 1231 | PRCD |
| 1232 | TNNT2 |
| 1233 | RABL3 |
| 1234 | HAVCR1 |
| 1235 | PRPF3 |
| 1236 | TOPORS |
| 1237 | SPATA7 |
| 1238 | MIR217 |
| 1239 | ARMC5 |
| 1240 | IL6ST |
| 1241 | ETS1 |
| 1242 | RP9 |
| 1243 | POMGNT1 |
| 1244 | RP2 |
| 1245 | DNASE1 |
| 1246 | LIF |
| 1247 | CDH2 |
| 1248 | FLG |
| 1249 | DLG4 |
| 1250 | LYVE1 |
| 1251 | TRAF6 |
| 1252 | MFGE8 |
| 1253 | ADRB1 |
| 1254 | NF1 |
| 1255 | IL22 |
| 1256 | COL4A5 |
| 1257 | GUCA1B |
| 1258 | CLRN1 |
| 1259 | FAM161A |
| 1260 | PCARE |
| 1261 | MIR196A2 |
| 1262 | OCRL |
| 1263 | TUG1 |
| 1264 | KIF11 |
| 1265 | MT-CYB |
| 1266 | MIR26A1 |
| 1267 | DCAF8 |
| 1268 | OXTR |
| 1269 | IDH3B |
| 1270 | APOH |
| 1271 | GJB1 |
| 1272 | EDA |
| 1273 | IFNA2 |
| 1274 | TNFAIP3 |
| 1275 | NR1H3 |
| 1276 | SDC3 |
| 1277 | BAX |
| 1278 | STAT5B |
| 1279 | ACVRL1 |
| 1280 | A2M |
| 1281 | SRSF6 |
| 1282 | EPAS1 |
| 1283 | EPHB4 |
| 1284 | TNFSF10 |
| 1285 | TP53INP2 |
| 1286 | MIR34B |
| 1287 | ITGA4 |
| 1288 | PSEN1 |
| 1289 | NDUFS2 |
| 1290 | SERPINB2 |
| 1291 | ATF3 |
| 1292 | PEPD |
| 1293 | GNRHR |
| 1294 | NEK2 |
| 1295 | ARL2BP |
| 1296 | NAT2 |
| 1297 | FOXP1 |
| 1298 | MIR181B2 |
| 1299 | NES |
| 1300 | WT1 |
| 1301 | IGF2BP1 |
| 1302 | TRIM32 |
| 1303 | IDH3A |
| 1304 | PDE6G |
| 1305 | KIAA1549 |
| 1306 | SEMA4A |
| 1307 | SLC7A14 |
| 1308 | SNRNP200 |
| 1309 | CPB2 |
| 1310 | PCNA |
| 1311 | KRT16 |
| 1312 | YAP1 |
| 1313 | MST1R |
| 1314 | ADAM15 |
| 1315 | NR5A2 |
| 1316 | CCN4 |
| 1317 | MIR24-2 |
| 1318 | PRKG1 |
| 1319 | FLT4 |
| 1320 | SMAD7 |
| 1321 | BAMBI |
| 1322 | FLNA |
| 1323 | P2RX7 |
| 1324 | CYP1A1 |
| 1325 | EFEMP2 |
| 1326 | NLRP1 |
| 1327 | GRHL3 |
| 1328 | GHRH |
| 1329 | FMR1 |
| 1330 | KRT19 |
| 1331 | VAMP2 |
| 1332 | EFNB2 |
| 1333 | POLA1 |
| 1334 | CNGA1 |
| 1335 | MAK |
| 1336 | PRPF4 |
| 1337 | CDHR1 |
| 1338 | RGR |
| 1339 | FSCN2 |
| 1340 | KLHL7 |
| 1341 | KIZ |
| 1342 | RP1 |
| 1343 | HGSNAT |
| 1344 | NR1I2 |
| 1345 | BACH2 |
| 1346 | PRDX6 |
| 1347 | AMH |
| 1348 | DNAH8 |
| 1349 | CHIT1 |
| 1350 | LRG1 |
| 1351 | ERBB4 |
| 1352 | SLIT2 |
| 1353 | SLC6A4 |
| 1354 | BTC |
| 1355 | TXN |
| 1356 | DDR1 |
| 1357 | TGFBI |
| 1358 | HSP90B1 |
| 1359 | SOX9 |
| 1360 | KLK3 |
| 1361 | LGALS1 |
| 1362 | DRD4 |
| 1363 | CD80 |
| 1364 | MANF |
| 1365 | CSF1 |
| 1366 | ADAMTSL1 |
| 1367 | GABPA |
| 1368 | CXCL6 |
| 1369 | MAP2K7 |
| 1370 | PRNP |
| 1371 | ASAH2 |
| 1372 | RPS20 |
| 1373 | CD248 |
| 1374 | HNP1 |
| 1375 | CXCL5 |
| 1376 | CARD14 |
| 1377 | NANOG |
| 1378 | HSPB3 |
| 1379 | EOLA1 |
| 1380 | CD33 |
| 1381 | C4BPA |
| 1382 | POU5F1P3 |
| 1383 | SMIM10L2B |
| 1384 | POU5F1P4 |
| 1385 | BTK |
| 1386 | PRDX2 |
| 1387 | NOS1AP |
| 1388 | DEFB4B |
| 1389 | DIH1 |
| 1390 | IL17B |
| 1391 | PDCD4 |
| 1392 | ANG |
| 1393 | LINC00641 |
| 1394 | DEFA3 |
| 1395 | GADD45A |
| 1396 | MIR23C |
| 1397 | ABCB6 |
| 1398 | TUBB4B |
| 1399 | SLC9A6 |
| 1400 | TNFSF13B |
| 1401 | IL24 |
| 1402 | PRRT2 |
| 1403 | DEFB104A |
| 1404 | LINC00692 |
| 1405 | HDAC2 |
| 1406 | CCN3 |
| 1407 | OXA1L |
| 1408 | DEFB104B |
| 1409 | MIR23B |
| 1410 | HSPB2 |
| 1411 | SMIM10L2A |
| 1412 | IL20 |
| 1413 | SLC2A4 |
| 1414 | WNT10B |
| 1415 | HLA-DQB1 |
| 1416 | BSCL2 |
| 1417 | HLA-DQA1 |
| 1418 | HOXA13 |
| 1419 | SEM1 |
| 1420 | MPZ |
| 1421 | PLAGL1 |
| 1422 | IL23R |
| 1423 | EIF2AK3 |
| 1424 | GAD1 |
| 1425 | SALL1 |
| 1426 | GAD2 |
| 1427 | TRPV4 |
| 1428 | SLC19A2 |
| 1429 | CDKAL1 |
| 1430 | HOXD13 |
| 1431 | GDAP1 |
| 1432 | DLX6 |
| 1433 | STAT4 |
| 1434 | MEFV |
| 1435 | RYR1 |
| 1436 | GLI3 |
| 1437 | PITX1 |
| 1438 | NEFL |
| 1439 | DARS2 |
| 1440 | RAB7A |
| 1441 | TTN |
| 1442 | MTTP |
| 1443 | AIRE |
| 1444 | GARS1 |
| 1445 | MT-ATP6 |
| 1446 | KIF1A |
| 1447 | IFNGR1 |
| 1448 | MPV17 |
| 1449 | BTRC |
| 1450 | HLA-DPB1 |
| 1451 | INPP5E |
| 1452 | SH3TC2 |
| 1453 | CCR1 |
| 1454 | IGHMBP2 |
| 1455 | PALB2 |
| 1456 | LEF1 |
| 1457 | CYP2C9 |
| 1458 | RETREG1 |
| 1459 | SORD |
| 1460 | IRF5 |
| 1461 | CAVIN1 |
| 1462 | FIG4 |
| 1463 | SBF2 |
| 1464 | BBS1 |
| 1465 | SHBG |
| 1466 | PSTPIP1 |
| 1467 | ZMPSTE24 |
| 1468 | VPS13B |
| 1469 | PRX |
| 1470 | SNRPN |
| 1471 | IL12A |
| 1472 | TAP2 |
| 1473 | WNK1 |
| 1474 | FXN |
| 1475 | GDF5 |
| 1476 | NDRG1 |
| 1477 | ICOSLG |
| 1478 | EGR2 |
| 1479 | CDKN1C |
| 1480 | TAP1 |
| 1481 | LITAF |
| 1482 | MGAM |
| 1483 | IL37 |
| 1484 | APOC3 |
| 1485 | MME |
| 1486 | SLC37A4 |
| 1487 | CCT5 |
| 1488 | PPY |
| 1489 | BLM |
| 1490 | RRM2B |
| 1491 | ITLN1 |
| 1492 | HERC2 |
| 1493 | IL17F |
| 1494 | NR1H4 |
| 1495 | LBR |
| 1496 | CLIP2 |
| 1497 | TNNT3 |
| 1498 | FKBP6 |
| 1499 | TLR7 |
| 1500 | VCP |
| 1501 | CDKN1B |
| 1502 | POLD1 |
| 1503 | NR0B2 |
| 1504 | MARS1 |
| 1505 | PON2 |
| 1506 | PYY |
| 1507 | GTF2I |
| 1508 | DYNC1H1 |
| 1509 | PI4KA |
| 1510 | MECP2 |
| 1511 | FREM2 |
| 1512 | MLH1 |
| 1513 | UBA2 |
| 1514 | BCL7B |
| 1515 | DNAJC30 |
| 1516 | COG2 |
| 1517 | ERCC6 |
| 1518 | BUD23 |
| 1519 | MVK |
| 1520 | IRGM |
| 1521 | CYP27B1 |
| 1522 | HBA2 |
| 1523 | SAMHD1 |
| 1524 | GLI1 |
| 1525 | KIT |
| 1526 | TREX1 |
| 1527 | EIF4H |
| 1528 | RET |
| 1529 | MTMR2 |
| 1530 | LMNB2 |
| 1531 | DPYD |
| 1532 | CARD9 |
| 1533 | GTF2IRD2 |
| 1534 | VPS37D |
| 1535 | ARID1B |
| 1536 | TPO |
| 1537 | GRP |
| 1538 | SERPINA12 |
| 1539 | RFC2 |
| 1540 | GTF2IRD1 |
| 1541 | CHD7 |
| 1542 | GNB3 |
| 1543 | MICA |
| 1544 | HARS1 |
| 1545 | METTL27 |
| 1546 | TMEM270 |
| 1547 | NPM1 |
| 1548 | CLEC7A |
| 1549 | SDHB |
| 1550 | SUFU |
| 1551 | DES |
| 1552 | S100A12 |
| 1553 | GNE |
| 1554 | CYP1B1 |
| 1555 | SETX |
| 1556 | LIG4 |
| 1557 | HSPG2 |
| 1558 | SMO |
| 1559 | TWIST1 |
| 1560 | XRCC4 |
| 1561 | TBX4 |
| 1562 | DNM2 |
| 1563 | HINT1 |
| 1564 | POLR1C |
| 1565 | TNNI2 |
| 1566 | ASCC1 |
| 1567 | NPHS2 |
| 1568 | NALCN |
| 1569 | ATRIP |
| 1570 | MAF |
| 1571 | GYG1 |
| 1572 | TTC7A |
| 1573 | CHEK2 |
| 1574 | PRDM12 |
| 1575 | OFC8 |
| 1576 | RHS |
| 1577 | LMS |
| 1578 | CNM6 |
| 1579 | SHFM4 |
| 1580 | SFMMP |
| 1581 | C10DUPq24 |
| 1582 | EEC3 |
| 1583 | MRK |
| 1584 | STHAG8 |
| 1585 | SLC11A1 |
| 1586 | SHFM1 |
| 1587 | MLTK |
| 1588 | KET |
| 1589 | DUP10q24 |
| 1590 | C17DUPp13.3 |
| 1591 | DUP17p13.3 |
| 1592 | HOX1J |
| 1593 | HOX4D |
| 1594 | NRAMP |
| 1595 | SHFD2 |
| 1596 | SHFLD |
| 1597 | SHFM1D |
| 1598 | SHFM6 |
| 1599 | SHSF3 |
| 1600 | TP73L |
| 1601 | ZAK |
| 1602 | SHFL1 |
| 1603 | SHFM5 |
| 1604 | MAP3K20 |
| 1605 | HOXD10 |
| 1606 | NRAMP1 |
| 1607 | SHFLD2 |
| 1608 | DLX5 |
| 1609 | SHFM3 |
| 1610 | SHFLD3 |
| 1611 | SHFM2 |
